# ULK1 promotes mitophagy via phosphorylation and stabilization of BNIP3

**DOI:** 10.1038/s41598-021-00170-4

**Published:** 2021-10-15

**Authors:** Logan P. Poole, Althea Bock-Hughes, Damian E. Berardi, Kay F. Macleod

**Affiliations:** 1 The Ben May Department for Cancer Research, The University of Chicago, Chicago, USA; 2 The Committee on Cancer Biology, The University of Chicago, Chicago, USA; 3 The Committee on Molecular Metabolism and Nutrition, The University of Chicago, Chicago, USA

**Keywords:** Cancer, Cell biology

## Abstract

UNC51-like kinase-1 (ULK1) is the catalytic component of the autophagy pre-initiation complex that stimulates autophagy via phosphorylation of ATG14, BECLN1 and other autophagy proteins. ULK1 has also been shown to specifically promote mitophagy but the mechanistic basis of how has remained unclear. Here we show that ULK1 phosphorylates the BNIP3 mitochondrial cargo receptor on a critical serine residue (S17) adjacent to its amino terminal LIR motif. ULK1 similarly phosphorylates BNIP3L on S35. Phosphorylation of BNIP3 on S17 by ULK1 promotes interaction with LC3 and mitophagy. ULK1 interaction also promotes BNIP3 protein stability by limiting its turnover at the proteasome. The ability of ULK1 to regulate BNIP3 protein stability depends on an intact “BH3” domain and deletion of its “BH3” domain reduces BNIP3 turnover and increases BNIP3 protein levels independent of ULK1. In summary ULK1 promotes mitophagy by both stabilization of BNIP3 protein and via phosphorylation of S17 to stimulate interaction with LC3.

## Introduction

Macroautophagy (commonly referred to as autophagy) is activated in cells in response to nutrient deprivation, including hypoxia and amino acid deprivation^1^. The induction of autophagy by nutrient deficiency is largely mediated at a post-translational level as a result of AMPK-mediated phosphorylation of S555 and other serines in the ULK1 kinase that makes up the catalytic core of the autophagy pre-initiation complex^2–5^. Conversely, ULK1 is inactivated in the presence of amino acids as a result of phosphorylation by mTORC1 on S757^5^. ULK1, as part of the autophagy pre-initiation complex with FIP200, ATG13 and ATG101^6^, activates the downstream autophagy initiation complex through phosphorylation of Beclin1 and ATG14^7,8^, in addition to other substrates thereby enhancing VPS34 activity and phagophore membrane formation at ER-mitochondrial junctions^5^. ULK1 also phosphorylates FIP200, ATG13 and ATG101 and autophosphorylates as part of the pre-initiation complex. Defining the phosphorylation consensus site for ULK1 has permitted the identification of other substrates involved in autophagy, including AMBRA1 and VPS34^9^, in addition to novel targets like phosphofructokinase (PFK1) and STING that play less direct roles in autophagy^10,11^.

In addition to promoting increased general autophagy, ULK1 has also been implicated in selective autophagy and in the induction of mitophagy in particular^2–4,12,13^. In response to hypoxia for example, ULK1 was previously shown to interact with and phosphorylate the FUNDC1 mitochondrial cargo receptor to stimulate mitophagy^12^. ULK1 phosphorylated FUNDC1 on S17 adjacent to its LC3-interacting region (LIR) to promote LC3 interaction and mitophagic flux^12^. Similarly, ULK1 was shown to stimulate mitophagy induced in mammalian cells by BCL2-L-13, and while this was associated with phosphorylation of BCL2-L-13 on S252 adjacent to its LIR motif, the role of ULK1 in executing this specific phosphorylation event on BCL-L-13 was not pinned down^13^. As we have reported previously, there are multiple mechanisms to promote mitophagy, including Parkin/PINK1 mediated mechanisms and via BNIP3 and BNIP3L (NIX) dependent pathways^14,15^. Why the cell relies on such a diverse range of mitophagy modulators is not yet clear although we have suggested that this allows the cell to respond to diverse stresses that impinge upon mitochondrial function^14^.

Interestingly, BNIP3 and BNIP3L have both been reported previously to be phosphorylated on serine residues adjacent to their LIR motif in manners that increase the affinity and specificity of binding to different LC3 family members^16,17^. However, the kinase responsible for these phosphorylation events was not previously shown. Here, we report for the first time that ULK1 phosphorylates BNIP3 on S17 and BNIP3L on S35 adjacent to their respective LIR motifs. Furthermore, we show that this increases interaction with processed LC3B and promotes mitophagy induced by BNIP3 over-expression. This is further influenced by ULK1 due to the effect of ULK1 on increasing BNIP3 protein levels as a result of decreased proteasomal turnover. Here, we show that deletion of the BH3 domain renders BNIP3 protein resistant to proteasomal turnover and to the effects of ULK1 on its protein levels. We propose a model in which ULK1 binds to BNIP3 protecting it from proteasomal degradation while simultaneously phosphorylating BNIP3 on S17 to promote its interaction with LC3 and increase rates of mitophagy.

## Results

### Phosphorylation of BNIP3 and BNIP3L by ULK1

**Figure 1:**
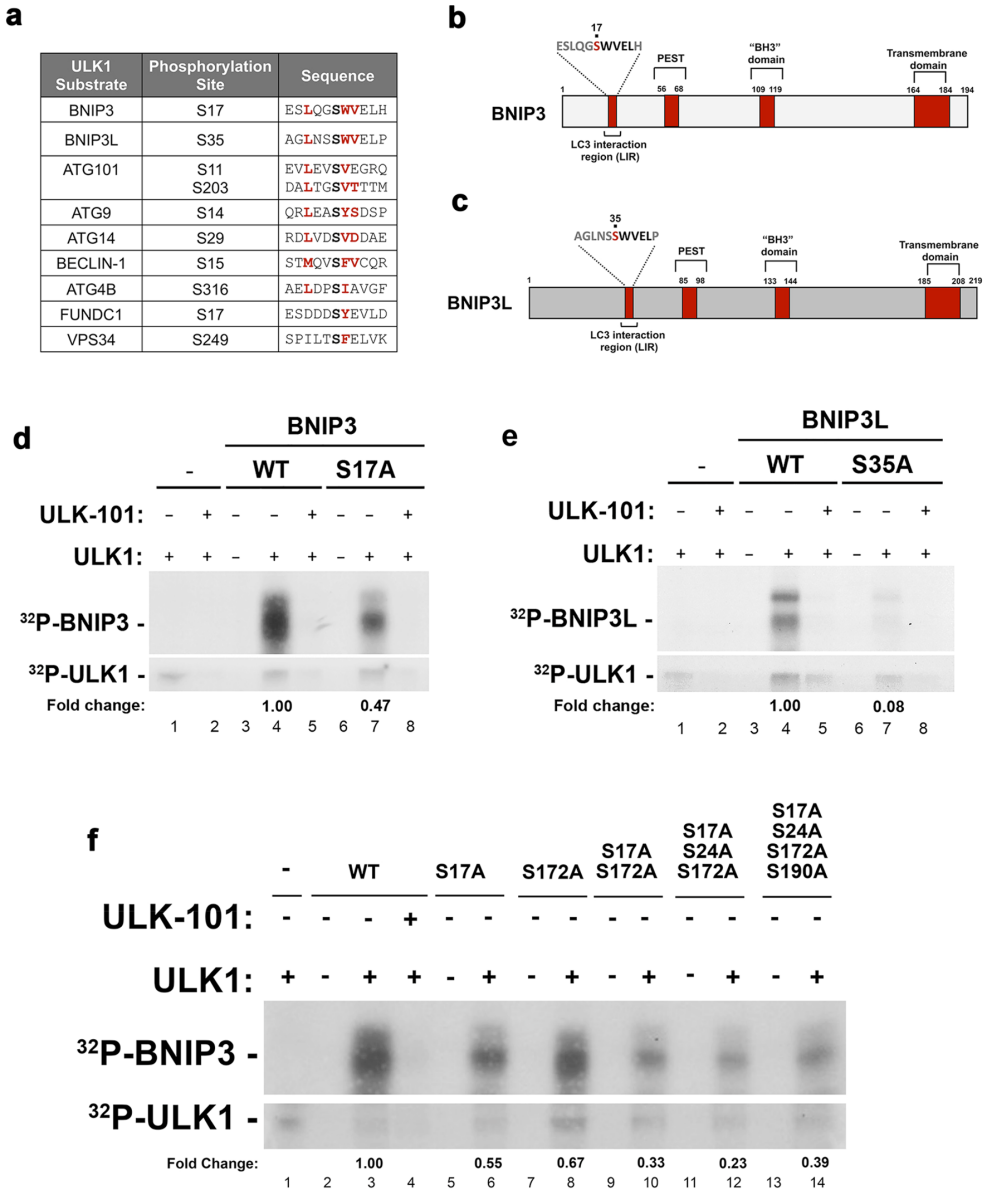
In vitro kinase assays show BNIP3 and BNIP3L are phosphorylated by ULK1 on S17 and S35 respectively. (**a**) Primary amino acid sequence alignment of putative ULK1 phosphorylation sites in BNIP3 and BNIP3L aligned to ULK1 phosphorylation sites in validated ULK1 substrates (ATG101, ATG9, ATG14, BECLIN1, ATG4B, FUNDC1, VPS34). (**b**) Cartoon illustrating key domains in BNIP3. (**c**) Cartoon illustrating key domains in BNIP3L. (**d**) In vitro kinase assay testing the ability of recombinant ULK1 kinase to phosphorylate recombinant BNIP3 (lanes 3–5) or BNIP3 mutated to S17A (lanes 6–8) in the presence or absence of ULK-101 to inhibit ULK1 kinase activity (lanes 2, 5, 8) and using ULK1 autophosphorylation as a control for ULK1 activity (lower panel). Fold change in phosphorylation for the mutant is shown relative to WT. (**e**) In vitro kinase assay testing the ability of recombinant ULK1 kinase to phosphorylate recombinant BNIP3L (lanes 3–5) or BNIP3L mutated to S35A (lanes 6–8) in the presence or absence of ULK-101 to inhibit ULK1 kinase activity (lanes 2, 5, 8) and using ULK1 autophosphorylation as a control for ULK1 activity (lower panel). Fold change in phosphorylation for the mutant is shown relative to WT. (**f**) In vitro kinase assay testing the ability of recombinant ULK1 kinase to phosphorylate different mutant forms of BNIP3. Fold changes in phosphorylation for each mutant is shown relative to WT.

Both BNIP3 and BNIP3L have previously been reported to be phosphorylated on serine 17 and 24 for BNIP3^18^ and on serine 34 and 35 for BNIP3L^17^, but the kinase responsible for these phosphorylation events has not been identified. Interestingly, S17 and S35 in BNIP3 and BNIP3L respectively map adjacent to critical tryptophan residues at W18 and W36 in each protein that form part of the LC3 interacting region (LIR) required for the ability of BNIP3 and BNIP3L to bind processed LC3 family members^17,19,20^. Serine residues adjacent to the LIR motif of other LC3 interacting proteins have been shown to be phosphorylated by ULK1, the core catalytic component of the autophagy pre-initiation complex^7,8,21^. The optimal amino acid sequence for an ULK1 phosphorylation site includes a preference for serine (S) over threonine (T) at the phosphorylation site, leucine (L) or methionine (M) at position − 3 and an aliphatic or aromatic amino acid, such as phenylalanine (F) or tryptophan (W) at positions + 1 and + 2^9^. When we aligned the primary amino acid sequence around S17 in BNIP3 and S35 in BNIP3L with validated ULK1 substrates and with the published optimal sequence for ULK1 phosphorylation sites^9^, we observed that amino acid sequences around S17 in BNIP3 and S35 in BNIP3L show strong sequence similarity to sites of phosphorylation by ULK1 (Fig. 1a). Specifically, both BNIP3 and BNIP3L have a leucine (L) at position − 3, serine (S) at position 0 and tryptophan (W) and valine (V) at positions + 1 and + 2 (Fig. 1a) with the W (+ 1) and V (+ 2) forming part of their LIR motifs (Fig. 1b,c).

To examine whether BNIP3 and/or BNIP3L are phosphorylated by ULK1, we performed in vitro kinase assays with recombinant BNIP3 and BNIP3L protein, incubated with recombinant ULK1 and ^32^P-g-ATP, in the presence or absence of the ULK1 inhibitor, ULK-101^22^. ULK1 strongly phosphorylated BNIP3 and BNIP3L in vitro and this phosphorylation was inhibited by ULK-101 (Fig. 1d, lanes 4 and 5; Fig. 1e, lanes 4 and 5). ULK1 autophosphorylates in vitro in a manner inhibited by ULK1-101 (Fig. 1d, lanes 1 and 2; Fig. 1e, lanes 1 and 2) that provides a useful internal control for ULK1 activity in this assay. Mutation of S17 in BNIP3 to alanine (S17A) or S35 in BNIP3L to alanine (S35A) decreased phosphorylation of BNIP3 (0.47 fold) and BNIP3L (0.08 fold) by ULK1 (Fig. 1d, lane 7; Fig. 1e, lane 7). The S17A mutation in BNIP3 did not decrease phosphorylation by ULK1 as effectively as the S35A mutation in BNIP3L in this recombinant in vitro assay but mutation of additional serine residues in BNIP3 did not identify any further putative ULK1 phosphorylation sites (Fig. 1f). In summary, we have identified putative ULK1 phosphorylation sites in BNIP3 and BNIP3L at S17 and S35 respectively.

### ULK1-mediated phosphorylation of BNIP3 on S17 promotes its interaction with LC3B and mitophagy

**Figure 2:**
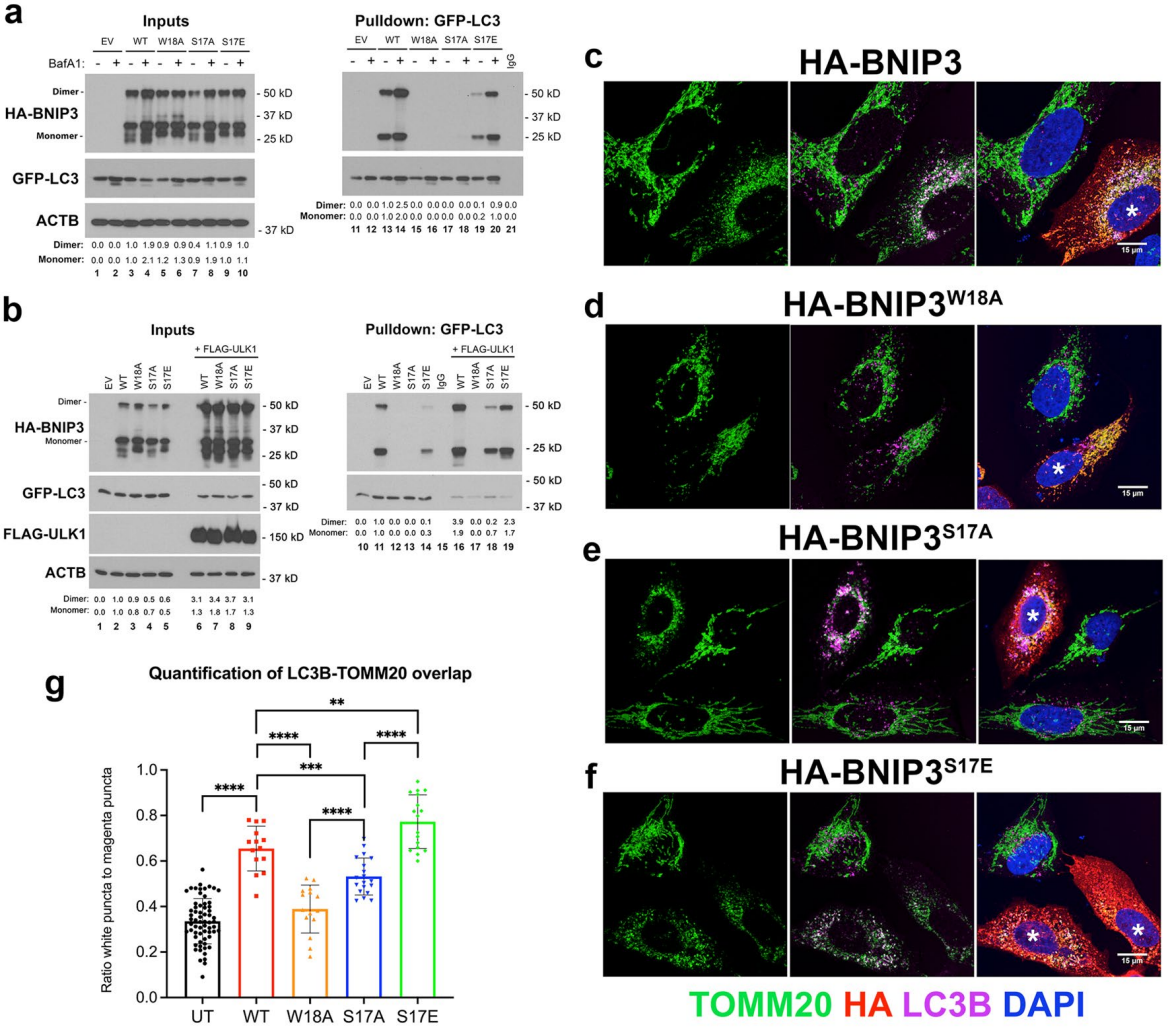
Mutation of S17 in BNIP3 modulates its LC3B interaction and mitophagy. (**a**) Pulldown of GFP-LC3 stably expressed in HEK-293 T cells with transiently expressed HA-BNIP3 (WT) and different HA-BNIP3 mutants (W18A, S17A, S17E) or empty vector (EV) control, in the presence or absence of 100 nM bafilomycin A_1_. Inputs to the pulldown are shown on the left and the result of the pulldown on the right. Fold changes in protein levels of BNIP3 dimer and BNIp3 monomer are shown relative to WT. (**b**) Pulldown of GFP-LC3 with HA-BNIP3, as described in (**a**), in the presence (lanes 6–9, 16–19) or absence (lanes 1–5, 10–15) of exogenous FLAG-ULK1. Fold change in protein levels of BNIP3 dimer and BNIp3 monomer are shown. Fold changes in protein levels of BNIP3 dimer and BNIp3 monomer are shown relative to WT. (**c**–**f**) Immunofluorescent staining for TOMM20 (green, mitochondria), LC3B (magenta, autophagosomes), HA-BNIP3 (red) and DAPI (blue) in U2OS cells transiently expressing HA-BNIP3 (**c**), HA-BNIP3^W18A^ (**d**), HA-BNIP3^S17A^ (**e**) or HA-BNIP3^S17E^ (**f**). Cells expressing exogenous HA-BNIP3 are asterisked (*) and LC3B/TOMM20 overlap is detected as white puncta (green and magenta overlap). (**g**) Quantification using Image J of LC3B/TOMM20 overlap per cell for at least at least 15 cells per field for each of the different forms of BNIP3 compared to cells not expressing BNIP3. Data were statistically analyzed as described in “Materials and methods” section. All data are shown as the mean ± s.e.m. Values of *p* ≤ 0.05 are considered significant. *p ≤ 0.05; **p ≤ 0.01; ***p ≤ 0.001; ****p ≤ 0.0001.

To examine how mutation of S17 affected BNIP3-dependent mitophagy, we mutated the putative ULK1 phosphorylation site at S17 to either alanine (S17A) to block ULK1 phosphorylation of BNIP3, or to glutamic acid (S17E) to mimic ULK1 mediated phosphorylation of BNIP3 and assessed how this affected the interaction of BNIP3 with LC3B. Mutation of S17 to alanine diminished interaction of BNIP3 with GFP-LC3B (Fig. 2a, lanes 17 and 18) compared to wild-type BNIP3 (Fig. 2a, lanes13 and 14) and to a similar extent as mutation of the critical W18 residue in the BNIP3 LIR motif to alanine (Fig. 2a, lanes 15 and 16) indicating that inhibiting phosphorylation of BNIP3 on S17 was sufficient to block its interaction with LC3B. Mutating S17 to glutamic acid to mimic phosphorylation resulted in apparently lower binding to LC3B than wild-type (Fig. 4a, lane 19 compared to lane 13). However, treatment of cells with 100 nM bafilomycin A_1_ to block autophagic turnover resulted in a more significant 9.0 fold increase in binding of BNIP3^S17E^ to LC3B (Fig. 2a, lane 20 compared to lane 19) than the 2.5 fold effect of bafilomycin A_1_ on wild-type BNIP3 (Fig. 2a, lane 14 compared to lane 13), suggesting that the S17E mutation promotes mitophagic flux.

We next assessed how ULK1 influenced the interaction of BNIP3 with LC3B (Fig. 2b). Interestingly, we noted that over-expressing FLAG-ULK1 increased the overall levels of expression of all BNIP3 forms (WT, W18A, S17A, S17E) examined (Fig. 2b, lanes 6–9 compared to lanes 2–5) suggesting that ULK1 was modulating BNIP3 protein levels. ULK1 over-expression also increased the interaction of wild-type BNIP3 with GFP-LC3B (Fig. 2b, lane 16 compared to lane 11) although this could be attributed to increased BNIP3 levels since the increased interaction is proportionate to the relative increase in BNIP3 protein levels. ULK1 over-expression had no effect on the failure of the W18A mutant to interact with GFP-LC3B (Fig. 2b, lane 17 compared to lane 12) but did modestly increase binding of the S17A mutant to GFP-LC3B (Fig. 2b, lane 18 compared to lane 13). ULK1 also increased the interaction of the S17E mutant with GFP-LC3 (Fig. 2b, lane 19 compared to lane 14). These results show that ULK1 both increases BNIP3 protein levels and increases the interaction of BNIP3 with LC3B.

**Figure 3:**
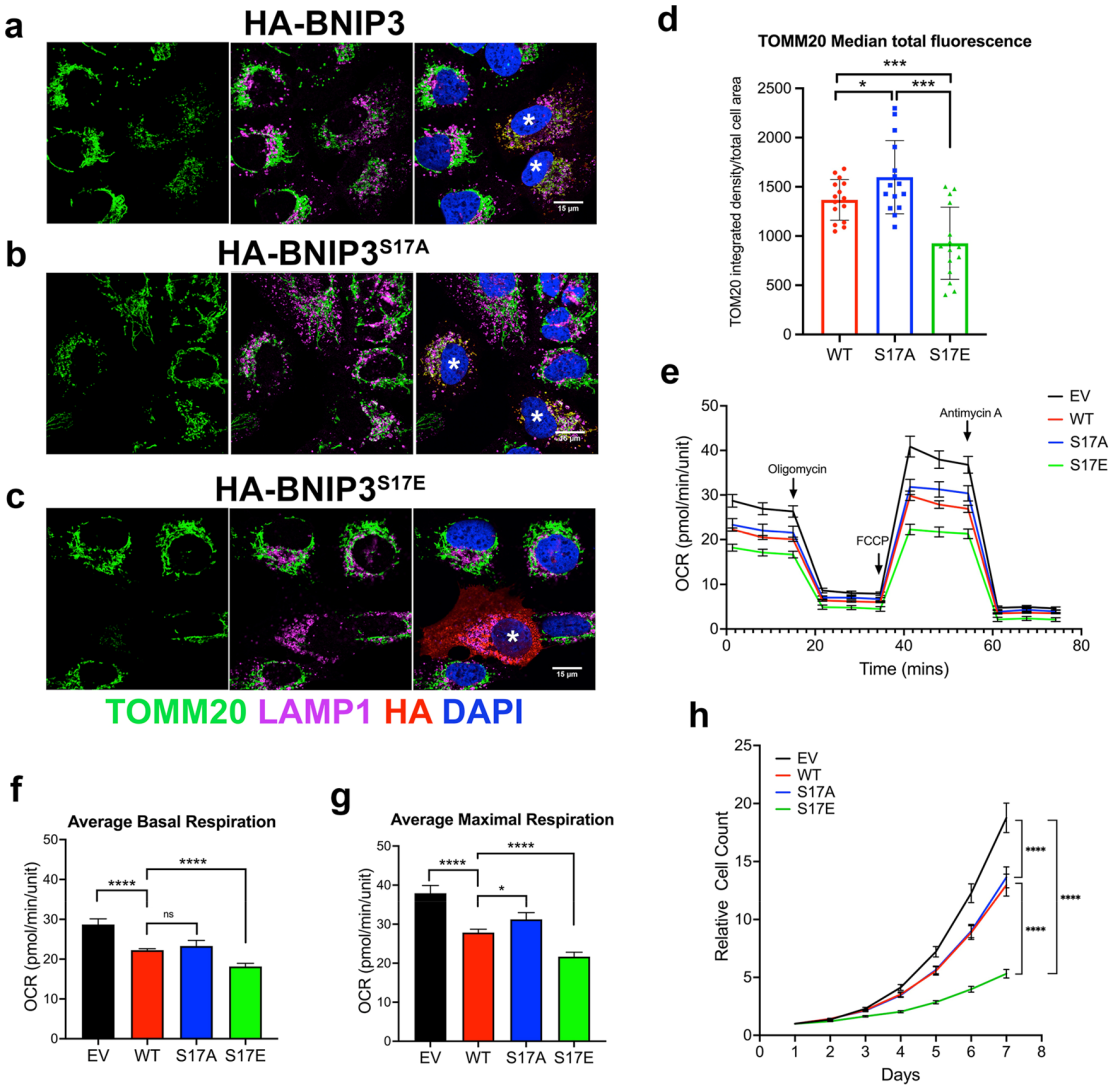
Phosphorylation of S17 promotes mitophagy, reduces oxygen consumption and decreases cell growth. (**a**–**c**) Immunofluorescent staining for TOMM20 (green, mitochondria), LAMP1 (magenta, lysosomes), HA-BNIP3 (red) and DAPI (blue) in U2OS cells transiently expressing HA-BNIP3 (**a**), HA-BNIP3^S17A^ (**b**) or HA-BNIP3^S17E^ (**c**). Cells expressing exogenous HA-BNIP3 (WT, S17A, S17E) are asterisked (*) and LAMP1/TOMM20 overlap is detected as white puncta (green and magenta overlap). (**d**) Quantification using Image J of total TOMM20 staining per cell per cell for at least at least 15 cells per field for each of the different forms of BNIP3 (WT—red, S17A—blue, S17E—green). (**e**) Oxygen consumption rate (OCR) of U2OS cells stably expressing HA-BNIP3, HA-BNIP3^S17A^ or HA-BNIP3^S17E^ compared to empty vector control (EV—black). OCR was performed in 96 well plates with 12 samples per treatment group. Values of *p* ≤ 0.05 are considered significant. *p ≤ 0.05; **p ≤ 0.01; ***p ≤ 0.001; ****p ≤ 0.0001. (**f**) Quantification of basal respiration at time 0 of cells represented in (**e**). (**g**) Quantification of maximal respiration at time 47 min of cells represented in (**e**). Values of *p* ≤ 0.05 are considered significant. *p ≤ 0.05; **p ≤ 0.01; ***p ≤ 0.001; ****p ≤ 0.0001. (**h**) Growth rate of U2OS cells stably expressing HA-BNIP3, HA-BNIP3^S17A^ or HA-BNIP3^S17E^ compared to empty vector control (EV) over a 7-day period as determined by IncuCyte S3 imaging system in 96 well format with 12 samples per treatment group. Data were statistically analyzed as described in “Materials and methods” section. All data are shown as the mean ± s.e.m. Values of *p* ≤ 0.05 are considered significant. *p ≤ 0.05; **p ≤ 0.01; ***p ≤ 0.001; ****p ≤ 0.0001.

Imaging of LC3B and TOMM20 in U2OS^DBNIP3^ cells transiently over-expressing BNIP3 or BNIP3^W18A^, BNIP3^S17A^ or BNIP3^S17E^ (Fig. 2c–f), showed that wild-type BNIP3 increased overlap (white puncta) in staining between TOMM20-positive mitochondria (green) and LC3-positive puncta (magenta) compared to adjacent cells not expressing BNIP3 (Fig. 2c,g). Expression of exogenous BNIP3 was also associated with increased mitochondrial fragmentation and decreased overall TOMM20 staining (Fig. 2c) indicative of decreased mitochondrial mass due to increased mitophagy. As reported previously in other systems, the W18A mutant of BNIP3 which is unable to bind LC3 (Fig. 2a,b) as defective at promoting TOMM20 (green)/LC3B (magenta) overlap and few white overlapping puncta were detected (Fig. 2d,g). Consistent with protein interaction data (Fig. 2a), the S17A mutant caused LC3B puncta to accumulate but there was decreased overlap between TOMM20 and LC3 when BNIP3^S17A^ was expressed (Fig. 2e,g) compared to wild-type BNIP3 (Fig. 2c,g), although more than in cells expressing BNIP3^W18A^ (Fig. 2d,g). Similar to the W18A mutant the S17A mutant retained the ability to induce mitochondrial fragmentation (Fig. 2e). By contrast, the BNIP3^S17E^ mutant induced marked overlap between TOMM20 and LC3B (Fig. 2f,g), and more effectively than wild-type BNIP3 (Fig. 2c,g), and very strikingly reduced mitochondrial staining in cells, such that S17E expressing U2OS^DBNIP3^ cells had much lower mitochondrial staining (Fig. 2f). Similar findings were obtained when cells were stained with lysosomal marker LAMP1 and TOMM20 to examine mitochondrial turnover at the lysosome such that the S17E removed most mitochondria by mitophagy (Fig. 3c,d) and to a greater extent than wild-type (Fig. 3a,d) while the S17A mutant had diminished ability to promote mitophagy compared to wild-type (Fig. 3b,d). Together, these findings indicate that the S17E mutation that mimics ULK1 phosphorylation markedly increases LCB interaction and mitophagy while the S17A mutation that blocks ULK1 phosphorylation decreases mitophagy relative to wild-type BNIP3 but not as effectively as the W18A mutant.

We then examined how the S17 mutants affected mitochondrial respiration (Fig. 3e–g) and cell growth (Fig. 3h). As shown, wild-type BNIP3 (WT) repressed oxygen consumption of U2OS^DBNIP3^ cells (Fig. 3e, red line) compared to those expressing empty vector (EV) (Fig. 3e, black line), as expected if BNIP3 is inducing mitophagy and decreasing mitochondrial mass (Figs. 2c,g, 3a). The S17A mutant was modestly less effective than wildtype at decreasing oxygen consumption although the differences in basal O_2_ consumption were within the margin of error (Fig. 3e, blue line; Fig. 3f). By far the biggest effect on O_2_ consumption was seen when the S17E mutant was expressed (Fig. 3e, green line; Fig. 3f,g) with a marked reduction in both basal (Fig. 3f) and maximal (Fig. 3g) oxygen consumption as expected given the strong positive effect of the S17E mutant on mitophagy (Figs. 2f,g, 3c,d). Consistent with the effects of these mutants on respiration, we observed that the S17E mutant caused the most dramatic slowdown in cell growth in culture, with both wild-type and S17A decreasing growth compared to empty vector expressing cells (Fig. 3h). Taken together, these results indicate that phosphorylation of BNIP3 on S17 promotes the interaction of BNIP3 with LC3, increases mitophagy, lowers respiration and decreases cell growth.

### The “BH3” domain modulates BNIP3 protein stability

As shown in Fig. 2b, over-expressing FLAG-ULK1 appeared to increase levels of HA-BNIP3. To gain insight to how ULK1 activity might be modulating BNIP3 protein levels, we explored the effect of ULK1 on levels of different BNIP3 mutants. In addition to the S17A and S17E BNIP3 mutants described thus far, we also examined effects of ULK1 on the DBH3 mutant in which amino acids 109 to 119 are removed, the DPEST mutant in which amino acids 56 to 68 were removed, the G180A point mutant that is not able to dimerize and the DTMD mutant lacking amino acids 164 to 184 that encodes the transmembrane domain (TMD) of BNIP3. The BH3 domain in BNIP3 is very loosely conserved with only 2 amino acids out of 11 conserved residues that make up a consensus BH3 domain^23^. In addition, BNIP3 binds Bcl2 and Bcl-X_L_ via its amino terminus not via its “BH3” domain, remains able to promote mitophagy in the absence of the BH3 domain and promotes survival not cell death suggesting that the “BH3” domain more likely reflects the evolutionary origin of BNIP3 from Bcl2 family members as opposed to BNIP3 acting as a *bona fide* BH3-only protein^23–26^. The PEST domain in BNIP3 was originally identified^27^ based on sequence homology to other proteins targeted for degradation due to similar regions enriched in Proline (P), Glutamic acid (E), Serine (S) and Threonine (T) residues^28^. Mutation of G180 to alanine within the transmembrane domain of BNIP3 prevents dimerization but not integration of the monomer into the outer mitochondrial membrane (OMM)^29^. Finally, the DTMD mutant cannot integrate into the mitochondrial outer membrane, cannot dimerize and cannot promote mitophagy^14,27,30^.

**Figure 4:**
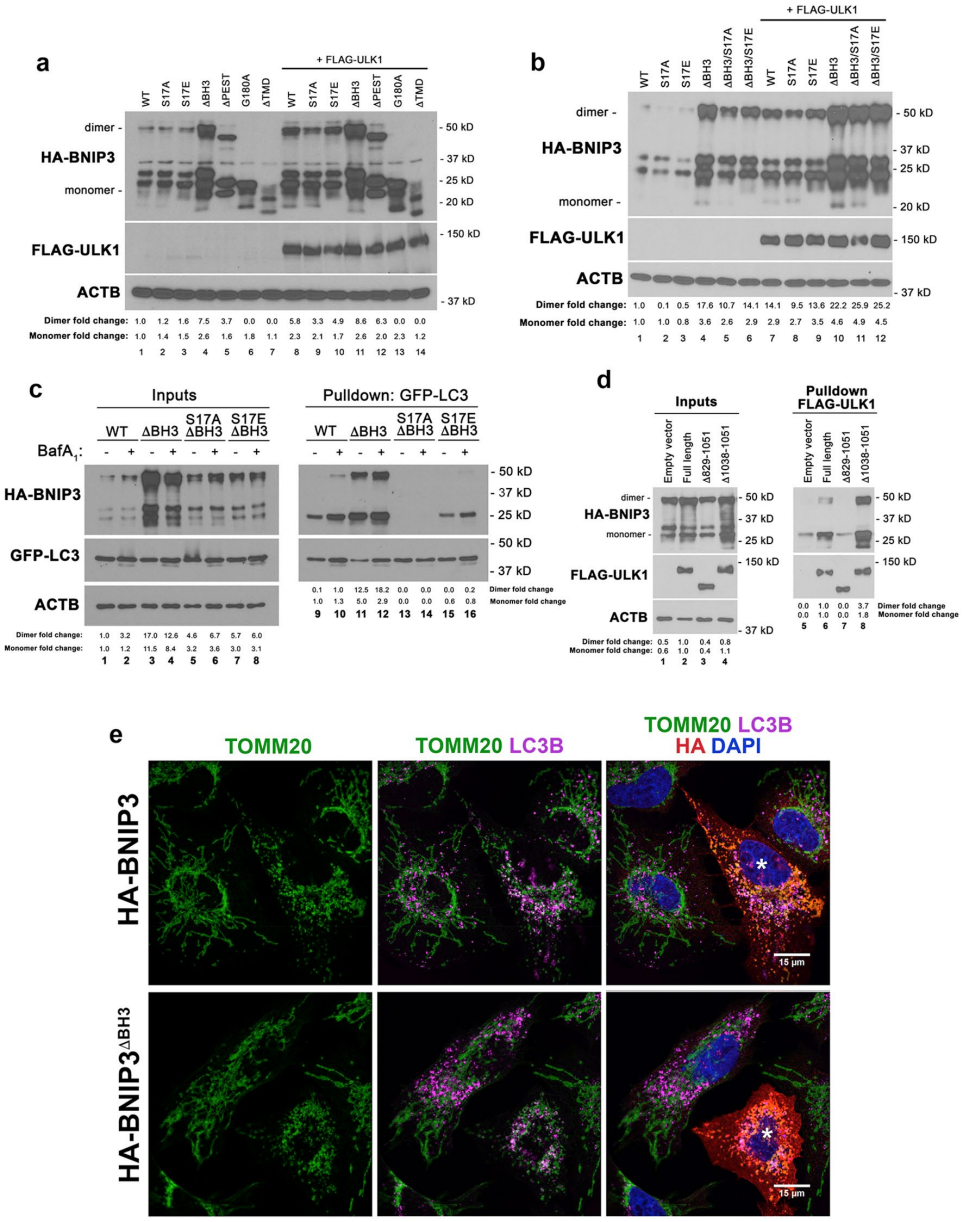
The BH3 domain promotes BNIP3 turnover in a manner inhibited by ULK1. (**a**) Western blot for different mutant forms of HA-BNIP3 (WT, S17A, S17E, DBH3, DPEST, G180A, DTMD) expressed in HEK-293 T cells in the presence (lanes 8–14) or absence (lanes 1–7) of exogenous FLAG-ULK1. Fold change in protein levels of BNIP3 dimer and BNIP3 monomer are shown relative to WT. (**b**) Western blot for HA-BNIP3 and different mutant forms (WT, S17A, S17E, DBH3, DBH3/S17A, DBH3/S17E) of HA-BNIP3 expressed in HEK-293 T cells in the presence (lanes 7–12) or absence (lanes 1–6) of exogenous FLAG-ULK1. Fold change in protein levels of BNIP3 dimer and BNIP3 monomer are shown relative to WT. (**c**) Pulldown of GFP-LC3 stably expressed in HEK-293 T cells with transiently expressed HA-BNIP3 (WT) and different HA-BNIP3 mutants (DBH3, S17A/DBH3, S17E/DBH3) in the presence or absence of 100 nM bafilomycin A_1_. Inputs to the pulldown are shown on the left and the result of the pulldown on the right. Fold change in protein levels of BNIP3 dimer and BNIP3 monomer are shown relative to WT. (**d**) Pulldown of FLAG-ULK1 stably expressed in HEK-293 T cells with transiently expressed HA-BNIP3 (WT) and different CTD mutant forms of FLAG-ULK1 (full-length, D829–1051, D1038–1051). Fold change in levels of BNIP3 dimer and BNIP3 monomer are shown relative to Full-length. (**e**) Immunofluorescent staining for TOMM20 (green, mitochondria), LC3B (magenta, autophagosomes), HA-BNIP3 (red) and DAPI (blue) in U2OS cells transiently expressing HA-BNIP3 (top panels) or HA-BNIP3^DBH3^ (bottom panels).

We compared levels of BNIP3 in the presence (Fig. 4a, lanes 8–14) or absence (Fig. 4a, lanes 1–7) of exogenous FLAG-ULK1 expression. Exogenous ULK1 enhanced the levels of wild-type BNIP3 (Fig. 4a, lane 8 compared to lane 1) consistent with data above (Fig. 2b). Similar to wildtype BNIP3, both the S17A and S17E mutants showed increased levels in the presence of exogenous ULK1 (Fig. 4a, lanes 9 and 10 compared to lanes 2 and 3) indicating that ULK1 could promote BNIP3 protein levels independent of its ability to phosphorylate serine 17. Similarly, the DPEST mutant was also increased in levels by exogenous ULK1 (Fig. 4a, lane 12 compared to lane 5) indicating that these sequences do not underlie the effect of ULK1 on BNIP3 protein levels. The G180A mutant also exhibited higher protein levels in the presence of exogenous ULK1 (Fig. 4a, lane 13 compared to lane 6) suggesting that dimerization was not required for BNIP3 levels to be modulated by ULK1. Interestingly, the DTMD mutant was minimally affected in levels by over-expression of exogenous ULK1 (Fig. 4a, lane 14 compared to lane 7) suggesting that the effect of ULK1 on BNIP3 levels relied on BNIP3 integration into the OMM. Apart from the DTMD mutant, the other mutant that behaved differently was the DBH3 mutant that exhibited high levels of expression even in the absence of FLAG-ULK1 (Fig. 4a, lane 4) compared to wild-type BNIP3 (Fig. 4a, lane 1) or any of the other BNIP3 mutants (Fig. 4a, lanes 2, 3, 5, 6, 7). Deletion of this region increased BNIP3 protein levels independent of ULK1 expression (Fig. 4a, compare lane 4 to lane 1) and exogenous ULK1 did not significantly increase levels of the DBH3 mutant further (Fig. 4a, lane 11 compared to lane 4). Taken together, these results suggested that sequences within the BH3 domain were promoting the proteasomal degradation of BNIP3 in a manner that could be suppressed by ULK1 and depended on BNIP3 integration into the OMM.

Combining the BH3 domain deletion with either the S17A or S17E point mutation increased levels of each compound mutant compared to the single S17A or S17E mutant, but not as much as the DBH3 deletion alone (Fig. 4b, lanes 5 and 6 compared to lane 4). The presence of ULK1 increased levels further for the compound S17A/DBH3 and S17E/DBH3 mutants to levels now comparable to that seen with the DBH3 mutant (Fig. 4b, lanes 11 and 12 compared to lane 10). That deletion of the BH3 domain in the S17A and S17E mutants stabilizes BNIP3 in the absence of ULK1 (Fig. 4b, lanes 5, 6 compared to lanes 2 and 3), but not as effectively as in the presence of ULK1 suggests that the S17A/DBH3 and S17E/DBH3 mutants are still getting turned over in the absence of ULK1 and that the S17 mutation also affects BNIP3 protein turnover. These results are consistent with the BH3 region of BNIP3 being key to the stabilization of BNIP3 protein by ULK1 and acting in concert with post-translational events at S17 of ULK1.

Deletion of the BH3 domain also increased the interaction of BNIP3 with LC3B (Fig. 4c, lanes 11 and 12 compared to lanes 9 and 10) which is likely explained by increased BNIP3 protein levels. Indeed, we observe that deletion of the BH3 domain increased overlap between TOMM20-positive mitochondria (green) and LC3B-positive autophagosomes (magenta) compared to wild-type BNIP3 (Fig. 4e). However, deletion of the BH3 domain failed to increase the interaction of the S17A mutant or the S17E mutant with LC3 (Fig. 4c, lanes 13–16) despite increased levels of both the S17A/DBH3 and S17E/DBH3 mutants (Fig. 4c, lanes 5–8) relative to wild-type BNIP3 (Fig. 4c, lanes 1–2). These results indicate that the phosphorylation status of S17 is dominant over the BH3 domain in determining interaction with LC3B.

Given that ULK1 promotes BNIP3 protein levels (Figs. 2b, 4a) and also that ULK1 phosphorylates BNIP3 on S17 (Fig. 1d), we examined whether ULK1 interacted with BNIP3 in pulldown experiments (Fig. 4d). Expression of wild-type HA-BNIP3 efficiently pulled down FLAG-ULK1 (Fig. 4d, lane 6) identifying BNIP3 as an additional autophagy protein that interacts with ULK1. The C-terminal domain mediates ULK1 interactions with numerous autophagy proteins, including ATG13^31^ and deletion of the C-terminal domain (CTD: amino acids 829–1051) of ULK1 was previously shown to generate a dominant negative form of ULK1 that retained auto-phosphorylation ability but reduced activity on known substrates (ATG13 for example) that inhibited LC3 processing and autophagy^31^. Our results indicate that BNIP3 is another autophagy protein that interacts with ULK1 via its CTD since deletion of the CTD of ULK1 (D829-1051), markedly decreased binding of BNIP3 to ULK1 (Fig. 4d, lane 7). Conversely, deletion of the very C-terminal 14 amino acids (1038–1051) of ULK1 markedly increased the interaction between BNIP3 and ULK1 (Fig. 4d, lane 8) suggesting that deletion of amino acids 1038–1051 removed sequences that bound other proteins that may be competing with BNIP3 for binding to ULK1. The data above supports a model in which ULK1 binds to BNIP3 via its CTD to stimulate BNIP3 phosphorylation on S17 and increase BNIP3 protein levels, with the overall effect of boosting rates of mitophagy.

### ULK1 promotes BNIP3 protein stability by preventing its proteasomal degradation

**Figure 5:**
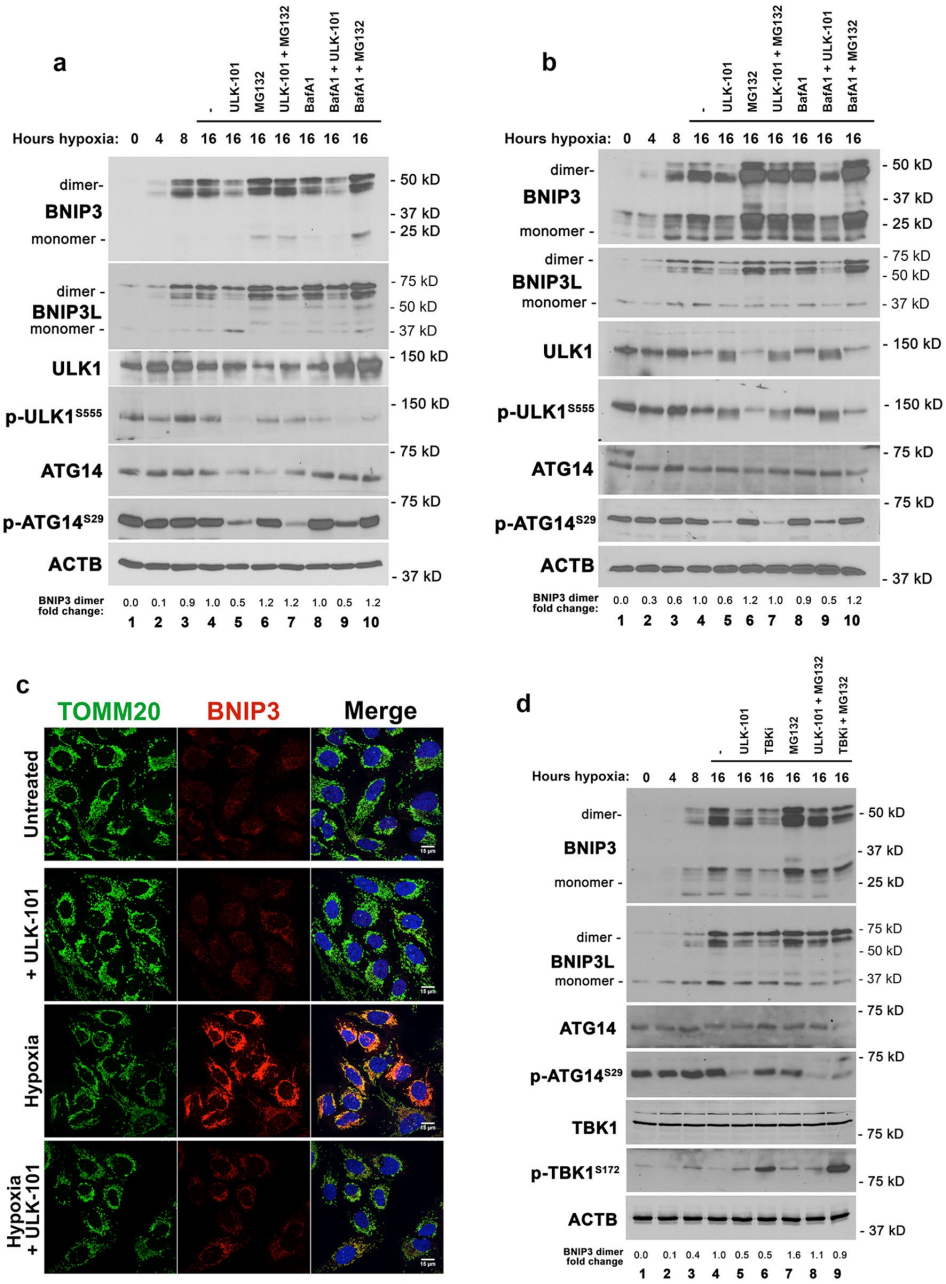
ULK1 inhibition limits BNIP3 accumulation under hypoxia. (**a**) Western blot for BNIP3, BNIP3L, ULK1, pS555-ULK1, ATG14, pS29-ATG14 and b-actin on whole cell lysates from U2OS cells exposed to hypoxia for 0, 4, 8 or 16 h (lanes 1–4) or for 16 h in the presence of vehicle control and/or ULK-101, MG132 or bafilomycin A1 (lanes 5–10). Fold change in protein levels of BNIP3 dimer are shown relative to the 16 h + hypoxia timepoint (lane 4). (**b**) Western blot for BNIP3, BNIP3L, ULK1, pS555-ULK1, ATG14, pS29-ATG14 and b-actin on. Whole cell lysates from Saos2 cells exposed to hypoxia for 0, 4, 8 or 16 h (lanes 1–4) or for 16 h in the presence of vehicle control and/or ULK-101, MG132 or bafilomycin A1 (lanes 6–10). Fold change in protein levels of BNIP3 dimer are shown relative to the 16 h + hypoxia timepoint (lane 4). (**c**) Immunofluorescent staining for TOMM20 (green, mitochondria), BNIP3 (red, autophagosomes) and DAPI (blue) in U2OS cells at atmospheric oxygen (Untreated), plus ULK1 inhibitor (+ ULK-101), 1% oxygen (hypoxia) or the combination of 1% oxygen plus the ULK1 inhibitor (hypoxia + ULK-101). (**d**) Western blot for BNIP3, BNIP3L, ATG14, pS29-ATG14, TBK1, p-TBK1S172 and b-actin on whole cell lysates from U2OS cells exposed to hypoxia for 0, 4, 8 or 16 h (lanes 1–4) or for 16 h in the presence of vehicle control and/or ULK-101, TBKi (MRT67307) or MG132 (lanes 5–9). Fold change in protein levels of BNIP3 dimer are shown relative to the 16 h + hypoxia timepoint (lane 4).

It was previously reported that ULK1 protein is induced and recruited to mitochondria by hypoxia^21^ and given that BNIP3 is also induced by hypoxia and localizes to mitochondria to promote hypoxia-induced mitophagy^26^, we speculated that ULK1 may be modulating the mitophagy functions of BNIP3 and BNIP3L during hypoxia. Both BNIP3 and BNIP3L were strongly induced by hypoxia such that at 8 h following the switch to 1% oxygen, both proteins were maximally expressed and their levels sustained through 16 h of hypoxia in both U2OS and Saos2 osteosarcoma cells (Fig. 5a, U2OS; Fig. 5b, Saos2—lanes 1–4). However, ULK1 protein levels were not significantly affected by hypoxia (Fig. 5a,b) and surprisingly nor was ULK1 activity since no change in the levels of phospho-S555 ULK1 was detected in either cell line examined (Fig. 5a,b; lane 4 compared to lane 1). Consistently, there was no difference in ULK1-mediated phosphorylation of ATG14 on S29 either, detected following 16 h at hypoxia compared to the zero timepoint (Fig. 5a,b; compare lane 4 to lane 1). These results suggest that ULK1 is constitutively active in these osteosarcoma lines.

When we inhibited ULK1 activity with ULK-101 however, we observed decreased phosphorylation of ULK1 on S555 and decreased phosphorylation ATG14 on S29 following growth in hypoxia for 16 h, despite no change in levels of either ULK1 or ATG14 protein levels (Fig. 5a,b; lane 5) consistent with ULK-101 effectively inhibiting ULK1 kinase activity. Interestingly, inhibition of ULK1 kinase activity markedly decreased levels of both BNIP3 and BNIP3L at 16 h of hypoxia (Fig. 5a,b, lane 5 compared to lane 4). ULK1 inhibition also markedly diminished the mitochondrial accumulation of BNIP3 in cells, as shown by co-staining for BNIP3 and TOMM20 (Fig. 5c). Addition of proteasomal inhibitor MG132 appeared to inhibit this effect of ULK-101 on BNIP3 and BNIP3L levels (Fig. 5a,b, lane 7 compared to lane 5) while treatment of cells with Bafilomycin A_1_ did not affect the ability of ULK-101 to decrease BNIP3 and BNIP3L levels (Fig. 5a,b, lane 9 compared to lane 5). This suggested that ULK1-101 promoted BNIP3 and BNIP3L turnover at the proteasome which conversely implies that ULK1 kinase activity is limiting proteasomal degradation of BNIP3 and BNIP3L.

Another mitophagy promoting kinase, TANK binding kinase-1 (TBK1) has also been shown to phosphorylate serine residues adjacent to the LIR motif of other cargo adaptor proteins, such as Optineurin (OPTN)^32–34^. To assess whether TBK1 could also modulate BNIP3 and BNIP3L, we examined the effect of the MRT67307 inhibitor of TBK1 (TBKi) on levels of BNIP3 and BNIP3L under hypoxia (Fig. 5d). Overall levels of TBK1 were not altered at all by hypoxia (Fig. 5d, lanes 1–4), or by inhibition of ULK1, TBK1 or the proteasome (Fig. 5d, lanes 5–9). Surprisingly, treatment of cells with MRT67307 (TBKi) resulted in increased detection of the p-TBK1^S172^ form of TBK1 (Fig. 5d, lanes 6 and 9). This has been reported previously^35^ and is explained as compensatory feedback activation of the TBK1 pathway in response to catalytic inhibition of TBK1. Thus, the increase in p-TBK1^S172^ levels is an indicator of decreased TBK1 activity following treatment with TBKi. Similar to the effect of inhibiting ULK1 (Fig. 5d, lane 5), TBK1 inhibition also decreased levels of BNIP3 and BNIP3L (Fig. 5d, lane 6). However, the effect of TBK1 on BNIP3 and BNIP3L levels was not associated with any change in ULK1 activity since treatment with the TBKi did not decrease p-ATG14^S29^ levels (lane 6), as was seen with the ULK1 inhibitor (lane 5). These results suggest that BNIP3 and BNIP3L may also be regulated by TBK1, in addition to their regulation by ULK1 and this is focus of ongoing studies.

**Figure 6:**
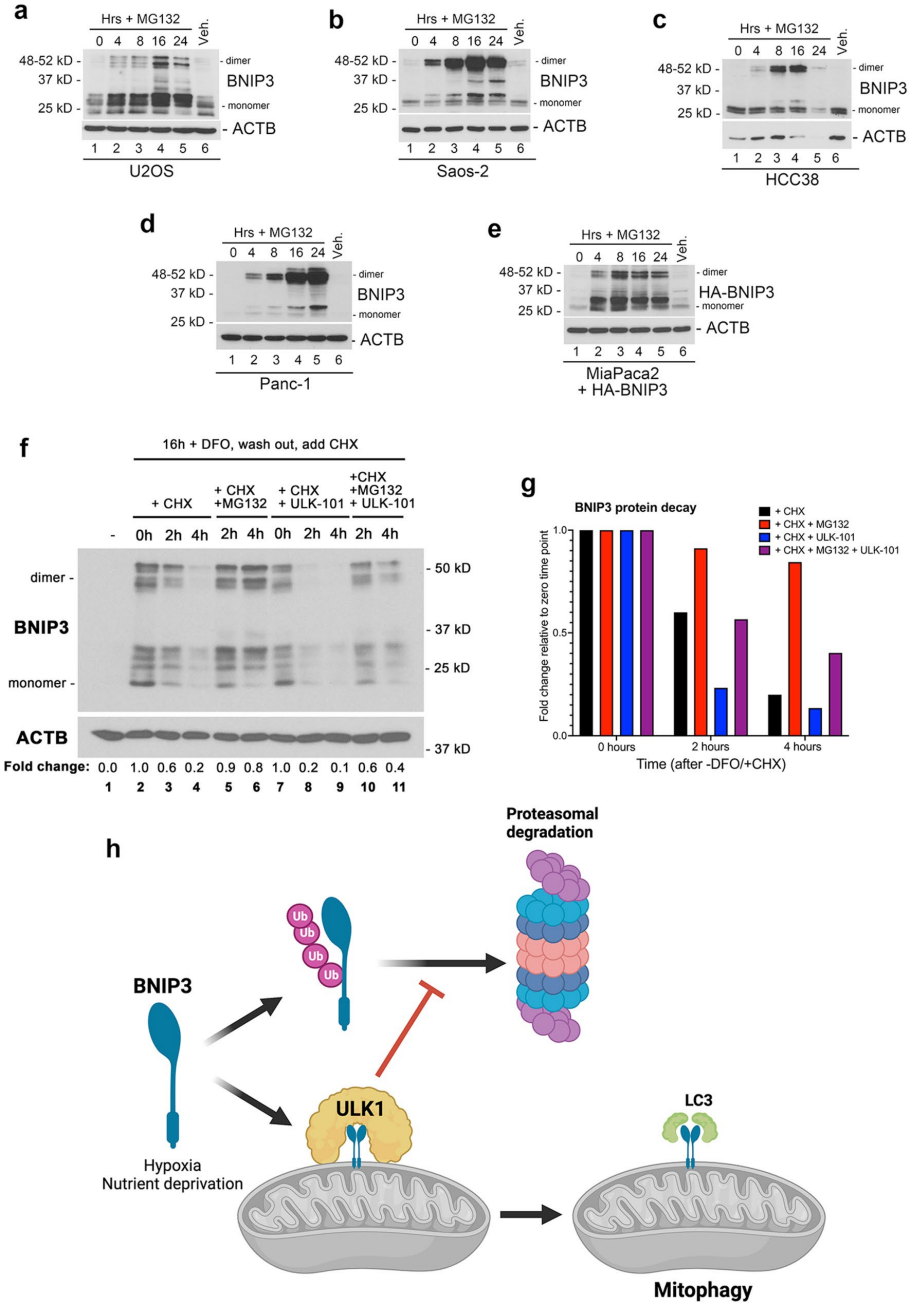
Proteasomal degradation of BNIP3 is inhibited by ULK1 kinase activity. (**a**–**d**) Western blot for endogenous BNIP3 in U2OS (**a**), Saos2 (**b**), HCC38 (**c**), Panc-1 (**d**) cells in response to MG132 treatment for 0, 4, 8, 16, 24 h. (**e**) Western blot for exogenous HA-BNIP3 in MiaPaca2 cells that are epigenetically silenced for endogenous BNIP3. (**f**) Western blot for endogenous BNIP3 in U2OS cells treated overnight with DFO to induce BNIP3, then washed out for DFO and immediately treated with cycloheximide (CHX) to block new protein synthesis and either MG132 to inhibit the proteasome and/or ULK-101 to inhibit ULK1 kinase activity. Fold change in BNIP3 dimer levels relative to lane 2 are shown. (**g**) Fold change in protein levels of BNIP3 dimer detected in (**g**) at 0, 2 and 4 h following release from DFO and addition of CHX are plotted relative to the 0 timepoint (lane 2). (**h**) Diagram summarizing the model for how ULK1 promotes BNIP3-dependent mitophagy—by both blocking its proteasomal turnover and phosphorylating BNIP3 to promote interaction with LC3B. This figure was generated in part using graphics from BioRENDER.

While analysis of BNIP3 and BNIP3L expression levels has generally focused on their transcriptional control by HIF1 and other transcription factors^26^, our data suggested that BNIP3 expression was strongly regulated at a post-translational level. This post-translational regulation was mediated via proteasomal degradation since BNIP3 protein levels were strongly increased in U2OS and Saos2 cells in response to MG132 treatment alone (Fig. 6a,b), and as observed in other cell lines (HCC38, Panc1) (Fig. 6c,d). Interestingly, MG132 rapidly (within 4 h) increased levels of exogenous HA-BNIP3 expressed in MiaPaca2 pancreatic cancer cells that are epigenetically silenced for endogenous BNIP3 (Fig. 6e), indicating that effects of MG132 on BNIP3 protein levels are not mediated indirectly through increased BNIP3 transcription. These results show that BNIP3 protein is being turned over at the proteasome in the absence of hypoxia or other physiological stresses, known to induce BNIP3 expression. That MG132 treatment did not further increase BNIP3 protein levels over that seen in cells grown at hypoxia (Fig. 5a,b, lane 6 compared to lane 4) suggested that hypoxia inhibits proteasomal turnover of BNIP3 protein. Taken together with observations described above that ULK1 inhibition decreased BNIP3 protein expression under hypoxia in a manner inhibited by MG132, suggests that hypoxia limits BNIP3 proteasomal degradation in a manner dependent on ULK1 activity.

To determine how ULK1 inhibition was increasing the rate of BNIP3 protein turnover, we examined endogenous BNIP3 levels following removal of iron chelator desferroxamine (DFO) that was added for 16 h to induce expression of BNIP3 at a transcriptional level via HIF-1a stabilization without inducing ROS, as would happen to cells switched out of 1% oxygen. Immediately following DFO removal, new protein synthesis was inhibited with cycloheximide (CHX) in the presence of either MG132 to inhibit proteasome activity, and/or ULK-101 to inhibit ULK1 kinase activity. Following removal of DFO in the presence of CHX, BNIP3 protein decayed rapidly such that by 4 h, it was barely detectable (Fig. 6f, lane 4 compared to lane 2; Fig. 6g). However, addition of MG132 to inhibit proteasomal activity prevented BNIP3 protein decay by 4 h and BNIP3 levels were similar to that seen at 0 h following removal of DFO in the presence of CHX (Fig. 6f, lane 6 compared to lane 2; Fig. 6g). Conversely, when ULK-101 was added, we observed a more rapid decline in BNIP3 protein levels such that by 2 h following removal of DFO in the presence of CHX, BNIP3 protein was barely detectable (Fig. 6f, lanes 7–9 compared to lanes 2–4; Fig. 6g). Combining MG132 and ULK-101 treatment protected against decay following removal of DFO in the presence of CHX indicating that ULK1 inhibition was causing proteasomal degradation of BNIP3 (Fig. 6f, lanes 10 and 11 compared to lanes 3, 4, 5, 6, 8, 9; Fig. 6g). These results indicate that ULK1 activity stabilizes BNIP3 protein levels by blocking its proteasomal degradation.

In summary, our work identifies BNIP3 (and BNIP3L) as an ULK1 substrate and shows that in addition to promoting the interaction of BNIP3 with LC3B and increasing mitophagy, ULK1 also promotes BNIP3 protein levels by inhibiting its proteasomal degradation (Fig. 6h). The BH3 domain of BNIP3 promoted the proteasomal turnover of BNIP3 and deletion of the BH3 domain caused BNIP3 protein to accumulate independent of ULK1 activity. These results also illustrate how phosphorylation of BNIP3 on S17 by ULK1 decreases oxygen consumption, as expected with less mitochondria, and reduces cell growth.

## Discussion

We show here that ULK1 phosphorylates BNIP3 on S17 adjacent to its LIR motif (amino acids 18–21) to promote interaction with LC3 and that ULK1 also increases BNIP3 protein levels by blocking its turnover at the proteasome. In this manner, ULK1 has a dual effect on BNIP3 that promotes mitophagy following its induction by hypoxia. ULK1 also phosphorylates BNIP3L on the cognate serine at position 35 adjacent to its LIR motif (amino acids 36 to 39). Both BNIP3 and BNIP3L have been previously reported to be phosphorylated on these serine residues^16,17^ but the identity of the kinase responsible was not known till now. Those previous studies also reported phosphorylation of S24 in BNIP3 and S34 in BNIP3L as modulating their interaction with LC3 family members. However, the primary amino acid sequence around these serine residues does not conform with the ULK1 consensus phosphorylation site^9^, and we do not detect loss of phosphorylation of BNIP3 by recombinant ULK1 when S24 is mutated (Fig. 1f) and mutation of S35 abolished all phosphorylation of BNIPL3 by ULK1 in vitro (Fig. 1d). These findings suggest that while ULK1 promotes LC3 binding by phosphorylating S17 in BNIP3 and S35 in BNIP3L, a different kinase is likely responsible for phosphorylating S24 and S34 in BNIP3 and BNIP3L respectively. TBK1 may be involved in such regulation of BNIP3 and BNIP3L since we show that inhibiting TBK1 causes a similar effect on their protein levels to inhibition of ULK1 (Fig. 5d), although the exact role of TBK1 in modulating BNIP3 and BNIP3L awaits further investigation. ULK1 has also been shown to phosphorylate FUNDC1 and BCL2-L-13 to promote their interaction with LC3 family members and mitophagy^13,21^. Together with BNIP3 and BNIP3L, that indicates ULK1 promotes mitophagy via four different mitochondrial cargo receptors and suggests that in addition to promoting general autophagy via phosphorylation of Beclin1 and ATG14, that ULK1 specifically upregulates mitophagy in nutrient stressed cells.

In addition to phosphorylating BNIP3 on S17 to promote LC3B interaction, ULK1 also increases BNIP3 protein levels (Figs. 2b, 4a,b) and conversely inhibition of ULK1 kinase activity with ULK-101 represses BNIP3 protein levels (Figs. 5a,b, 6f). ULK1 has a predicted molecular weight of 112 kD but generally migrates in SDS-PAGE with a molecular weight of approximately 150 kD, while BNIP3 is a fraction of the size at 21.4 kD, the carboxy terminal end of which is buried in the OMM^5,31,36,37^. Thus, binding of ULK1 to BNIP3 may protect it from proteasomal degradation simply due to size exclusion blocking access of E3 ubiquitin ligases to BNIP3 (Fig. 6g). Interestingly, previous work that we concur with, showed that amino acid deprivation and/or mTORC1 inhibition with Torin suppressed BNIP3 levels under hypoxia similar to what we observe with ULK1 inhibition^38^. Given that mTORC1 inhibits ULK1 activity, this may appear counter to our current findings. However, the effect of Torin was mediated via autophagy-dependent degradation of BNIP3^38^ whereas our current observations show ULK1 promoting BNIP3 protein levels by protecting it from proteasomal degradation. Thus, we propose that ULK1 modulates BNIP3 both positively by protecting it from proteasomal degradation in preparation for mitophagy, and negatively since BNIP3 is ultimately turned over by mitophagy. This is represented in the diagram in Fig. 6h in which initially ULK1 protects BNIP3 from proteasomal degradation and phosphorylates it to promote LC3 interaction and mitophagy, but subsequently as BNIP3-dependent mitophagy proceeds, BNIP3 is turned over with the mitochondria.

How then does BNIP3 get turned over by the proteasome? Both BNIP3 and BNIP3L are tail-anchored proteins, like many members of the Bcl2 super-family, that do not possess conventional mitochondrial-targeting signal peptides at their amino terminus, but instead rely on unique TMDs near their carboxy terminal end and key basic charged amino acids immediately after the TMD to integrate into the OMM^29,39–41^. A mitochondrial-associated degradation (MAD) system has been described similar to the endoplasmic reticulum associated degradation (ERAD) system in which the p97 AAA + ATPase induces retrotranslocation of proteins out of the OMM and presents them to the numerous E3 ubiquitin ligase complexes present at the OMM for degradation^42–45^ and indeed turnover of OMM proteins by the proteasome has been shown to be required to maintain mitochondrial function and cellular metabolism^46^. Parkin is amongst numerous different mitochondrial E3 Ub ligases implicated in maintaining the integrity of OMM protein function^47^. However, the E3 Ub ligases responsible for BNIP3 turnover are not known and will be the subject of future investigation.

Our work also showed that the BH3 domain of BNIP3 plays a role in turning over BNIP3 since its deletion markedly stabilized BNIP3 (Figs. 4a–c). As mentioned above, the BH3 domain of BNIP3 is very weakly conserved (2 out of 11 amino acids) with BH3 domains in other canonical BH3-only pro-apoptotic proteins like Bim or Puma, and indeed the BH3 domain of BNIP3 can be deleted with no loss of function in mitophagy^23–26^. Other functions for the BH3 domain in BNIP3 have been proposed including allowing BNIP3 to compete with Beclin1 for binding to Bcl-2 and Bcl-X_L_ thereby releasing Beclin1 to promote autophagy generally^16,37,48^. However, BNIP3 binds to Bcl-2 and Bcl-X_L_ primarily through its amino terminus, not through its BH3 domain, as revealed from the original yeast two-hybrid screen that identified BNIP3 as a Bcl-2 interacting protein^49^. Here, we show that deleting the BH3 domain increases BNIP3 protein levels and increases binding to LC3 setting forth a different model in which the “BH3 domain” limits mitophagy by promoting BNIP3 degradation by the proteasome. At this time, it is not clear how the BH3 domain promotes BNIP3 proteasomal degradation but there are two lysine residues at positions 111 and 112 in the BH3 domain of BNIP3 (amino acids 109 to 119) that could be subject to ubiquitination or sumoylation to promote BNIP3 turnover and dissecting how BNIP3 is turned over and the E3 Ub ligases responsible is the focus of future studies.

Finally, ULK1 is a core component of the autophagy pre-initiation complex and ULK1 inhibitors such as ULK-101 and others^9,22^ have been developed with a view to inhibiting autophagy as a cancer therapeutic approach. Indeed, ULK-101 preferentially killed KRas expressing tumor cells via inhibition of autophagic flux^22^. Moving forward, it will be informative to determine to what extent the beneficial effects of ULK-101 in preventing tumor growth are due to specific effects on mitophagy and BNIP3 levels as opposed to more general effects on overall autophagy.

## Materials and methods

### Site-directed mutagenesis

Site-directed mutagenesis was used for the generation of pLVX-IRES-hygro-HA-BNIP3 plasmids expressing mutant forms of BNIP3, and pcdna3 FLAG-ULK1 plasmids expressing mutant forms of ULK1. Primers were designed and recommended annealing temperatures were calculated using the NEBaseChanger website. Site-directed mutagenesis was then performed using the Q5 Site-directed Mutagenesis Kit (New England BioLabs).

### Cell culture

Human cell lines were all sourced from ATCC and maintained in a humidified CO_2_ incubator at 5% CO_2_ and 37 °C. U2OS, SaOS2, PANC-1, and HEK 293 T cell lines were cultured in Dulbecco’s Modified Essential Medium (DMEM) supplemented with 10% fetal bovine serum (FBS) and 1% penicillin/streptomycin. HCC38 cells were cultured in Roswell Park Memorial Institute (RPMI) media supplemented with 10% fetal bovine serum (FBS) and 1% penicillin/streptomycin. MiaPaca2 cells were cultured in DMEM supplemented with 10% FBS, 2.5% horse serum, and 1% penicillin/streptomycin. Cells treated with hypoxia were cultured in a humidified 37 °C hypoxia chamber at 5% CO_2_ and 1% O_2_. Cells were treated with drugs at the following concentrations: ULK-101 (5 μM), MG132 (10 μM), Bafilomycin A1 (0.1 μM), MRT67307 (100 nM), cycloheximide (10 μM), and deferoxamine (260 μM).

### Transfection

For the transient transfection of human cell lines, including HEK 293 T cells, cells were seeded onto 10 cm plates at a density of 1.0 × 10^6^ cells. The next day, 0.5 μg of pLVX-IRES-hygro-HA-BNIP3 plasmid and/or 1.0 μg of pcdna3 FLAG-ULK1 plasmid were added to Lipofectamine 3000 reagents at a 1:1 ratio (μg plasmid DNA:μL Lipofectamine 3000) in 0.5 mL of Opti-MEM media and allowed to incubate for 15 min at room temperature. After incubation, the solution was added to the 10 cm plates containing 8 mL of cell culture media. The plates were incubated in transfection media overnight, washed once with DPBS and returned to cell culture media. Cell lysates were harvested 36–48 h post-transfection.

### Generation of CRISPR/Cas9 BNIP3-KO cell lines

The BNIP3 locus was genetically deleted using CRISPR/Cas9 gene editing in HEK 293 T and U2OS cell lines to yield HEK-293T^DBNIP3^ cells and U2OS^DBNIP3^ cells respectively. BNIP3 CRISPR/Cas9 and HDR plasmids were purchased from Santa Cruz Biotechnologies (sc-400985 and sc-400985-HDR). Cell lines were transfected with 2 μg of each plasmid using Lipofectamine 3000 at a ratio of 2:1 Lipofectamine to DNA. After 24 h of transfection, media was changed and dual fluorescence of GFP and RFP was confirmed using the Incucyte S3 imaging system. Cells were selected 48–72 h post-transfection with 1 μg/mL puromycin and seeded sparsely onto 15 cm plates for clonal growth. Single clones were isolated using cloning cylinders. Hypoxia treated cell lysates were run on western blots and probed for BNIP3 to confirm absence of BNIP3 protein compared to control parental cells. The clones with confirmed deletion of BNIP3 were transiently transfected with Cre recombinase to remove the puromycin resistance genes and RFP. RFP deletion was then confirmed by western blot.

### Seahorse assays

U2OS^DBNIP3^ cells stably expressing pLVX-IRES-hygro-HA-BNIP3 mutants were seeded in Seahorse XF96 microplates at a density of 2 × 10^4^ cells/well. Following drug treatments, cells were rinsed with DPBS prior to the addition of 1X DMEM supplemented with 4.5 g/L glucose, 2 mM glutamine, and 1 mM sodium pyruvate, adjusted to a pH of 7.35. The Seahorse Cell Mito Stress Test was performed according to the manufacturer’s protocol using the Seahorse XF96 analyzer in the Biophysics Core at the University of Chicago. Data were normalized by cell density using Hoechst 33,342 nuclear counterstain and fluorescence quantification using a microplate reader. Normalized OCR data was then analyzed using Agilent Seahorse Wave software, version 2.6.1.53 (https://www.agilent.com/en/product/cell-analysis/real-time-cell-metabolic-analysis/xf-software/seahorse-wave-desktop-software-740897).

### Cell proliferation assays

U2OS^DBNIP3^ cells stably expressing NucLight-GFP were seeded at a density of 2 × 10^4^ cells per well in 6 well plates. Each condition was seeded in duplicate. The next day (D1), culture medium was changed, and the plates were placed in the Incucyte S3 Imaging system. The Incuyte S3 Imaging system counted fluorescent nuclei at 25 defined locations in each well once per day for D1 through D7. All counts were normalized to D1 values to account for seeding error.

### Protein extraction

Cells were seeded onto 10 cm plates at a density of 1.0 × 10^6^–1.5 × 10^6^ cells. Following experimental treatments, plates were scraped in DPBS containing protease and phosphatase inhibitors (0.5 mM PMSF, 1 μg/mL aprotinin, 1 μg/mL leupeptin, 1 mM Na_3_VO_4_). Cells were pelleted and resuspended in RIPA lysis buffer (10 mM Tris–HCl pH 8.0, 150 mM NaCl, 1% sodium deoxycholate, 0.1% SDS, 1% Triton X-100) containing a Roche PhosSTOP inhibitor cocktail in addition to the aforementioned protease and phosphatase inhibitors.

### Western blotting

Denatured protein (typically 75 μg) was loaded onto SDS-PAGE gels, followed by transfer to nitrocellulose (0.2 μm or 0.45 μm pore) or PVDF (0.45 μm pore) membranes. Membranes were blocked in 5% nonfat milk in TBS/0.05% Tween (TBS-T) for 30 min at room temperature for non-phosphorylated protein detection, and 5% BSA in TBS-T for 30 min at room temperature for phosphorylated protein detection. Membranes were cut as appropriate to allow multiple antibody stainings per transfer and incubated with primary antibodies overnight at 4 °C on a rocker in either 5% BSA/TBS-T or 5% nonfat milk/TBS-T depending on manufacturers’ protocols. The next day membranes were incubated with HRP-conjugated secondary antibody in 5% nonfat milk/TBS-T for 2 h at room temperature on a shaker. Proteins were visualized by chemiluminescence and exposure on X-ray film. Primary antibodies used for western blotting were as follows: BNIP3 (Cell Signaling cat # 44060), BNIP3L (Cell Signaling cat #12396), ULK1 (Cell Signaling cat #8054), p-ULK1^S555^ (Cell Signaling cat #5869), p-ULK1^S757^ (Cell Signaling cat #6888), ATG14 (Cell Signaling cat #5869), p-ATG14^S29^ (Cell Signaling cat #5869), TBK1 (Cell Signaling cat #3504), p-TBK1^S172^ (Cell Signaling cat. #5483), b-actin (Sigma Aldrich cat#A5441), FLAG (Cell Signaling cat #2368), HA (Cell Signaling cat #3724), GFP (Cell Signaling cat #2956). Western blots were repeated at least three times for each blot presented. Densitometric quantification of blots was performed using Image J and NIH image.

### Immunoprecipitation

Cells were seeded and collected as described above. Cell pellets were resuspended in NP-40 IP lysis buffer (50 mM Tris–HCl pH 7.5, 150 mM NaCl, 1 mM EDTA, 1% IGEPAL, 0.01% β-mercaptoethanol), and sonicated at 10% power for 5 s using a Fisher Sonic Dismembrator Model 500. GFP-tagged proteins were immunoprecipitated using GFP-Trap or control-Trap magnetic beads (Chromotek), and FLAG-tagged proteins were immunoprecipitated using anti-FLAG M2 magnetic or IgG-agarose beads (Sigma). Lysates were incubated on beads for 1 h at 4 °C on a rotator. Beads were transferred to a fresh Eppendorf tube for the final wash and resuspended in 2 × sample loading buffer (1:2:2 10 × SDS:5 × BPB: ddH_2_O).

### In vitro kinase assays

Recombinant GST-BNIP3 and GST-BNIP3L was produced in BL21 competent cells grown overnight and treated with IPTG for 4 h. Recombinant protein was purified using a Glutathione Sepharose 4B bead slurry and eluted via thrombin cleavage. In vitro kinase assays were performed using recombinant ULK1 (ThermoFisher PV6430), 5X Kinase Buffer A (ThermoFisher PV3189), recombinant BNIP3/BNIP3L, ATP (0.2 mM), ULK-101 (0.5 μM), and ATP [γ-32P] (PerkinElmer BLU002Z250UC). Assays were incubated at 37 °C for 30 min, diluted 1:1 in 2 × sample loading buffer, and boiled for 5 min. Samples were loaded onto an SDS-PAGE gel overnight, followed by gel drying and visualization by exposure on X-ray film.

### Immunofluorescence and confocal microscopy

U2OS^DBNIP3^ cells were grown on sterile glass coverslips in 6-well tissue culture plates overnight before transfection with HA-tagged BNIP3 mutants (see above). At 20 h post transfection, cells were treated with Bafilomycin A1 (100 nM) for 4 h. Cells were fixed for 15 min in 4% paraformaldehyde at RT, and 10 min in ice cold methanol at − 20 °C. Coverslips were incubated in 0.1% Saponin in PBS for 10 min, then blocked in 10% goat serum in 0.05% TBS-T for 1 h. Coverslips were then incubated with primary antibodies in 10% goat serum in TBS-T for 1 h at RT. Anti-TOMM20 (Abcam, ab56783, 1:200), anti-LC3B (Cell Signaling, 3868S, 1:200), anti-HA-Tag (Bethyl, A190-106A, 1:200), anti-LAMP1 (Abcam, ab25245, 1:200), anti-ULK-1 (Novus, NBP2-56576, 1:200), anti-BNIP3 (Cell Signaling, 44060, 1:200). Coverslips were washed in TBS-T for 3 × 5 min, followed by incubation with Alexa Fluor conjugated secondary antibodies (Thermo Fischer Scientific, 1:1000) for 1 h at RT. Coverslips were washed in TBS-T for 3 × 5 min and mounted with 10μL ProlongGold containing DAPI (Thermo Fisher, P36931). Slides were allowed to cure for 24 h in the dark at RT, with subsequent storage at 4 °C. Imaging was performed using the Leica TCS SP8 laser scanning confocal microscope in the Integrated Microscopy Core Facility at the University of Chicago. All images were collected using a 63X oil-immersion objective. Ten representative images per sample were obtained.

### Statistical analysis

All statistical analyses were carried out using GraphPad Prism of raw data. The data were analyzed using two-way ANOVA with Tukey’s post-test with a 95% confidence interval for data sets involving single parameters or single groups of data. Other datasets involving comparisons amongst multiple groups used Wilcoxon rank sum analyses with a 95% confidence interval. Data are shown as the mean ± s.e.m. Values of *p* ≤ 0.05 are considered significant. *p ≤ 0.05; **p ≤ 0.01; ***p ≤ 0.001; ****p ≤ 0.0001.

## Supplementary Information

Supplementary Information.
