# Supplementary figures and images for: ULK1 promotes mitophagy via phosphorylation and stabilization of BNIP3
Source: Sci Rep. 2021 Oct 15;11:20526. doi: 10.1038/s41598-021-00170-4 (PMC8519931; doi:10.1038/s41598-021-00170-4)

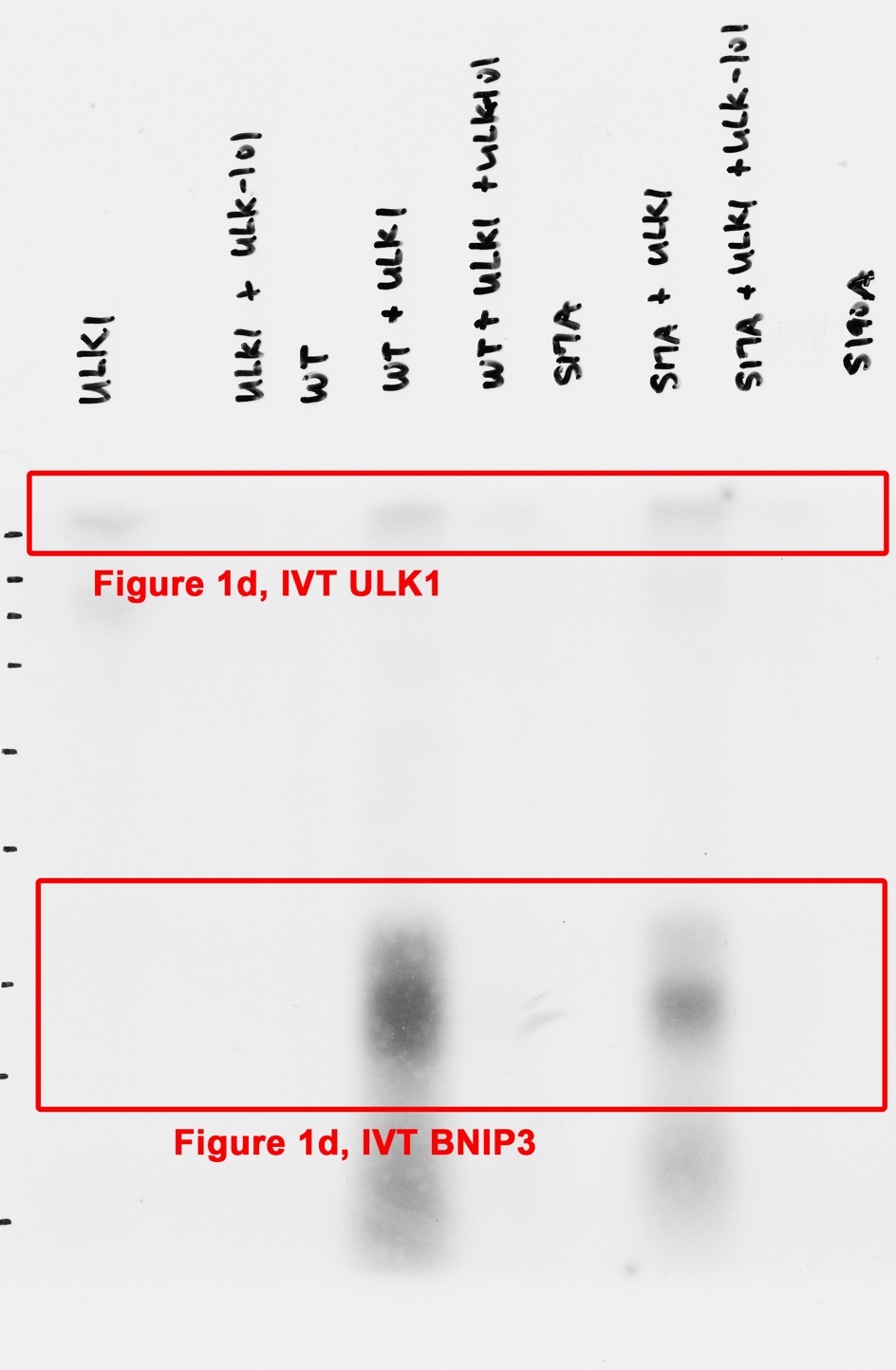


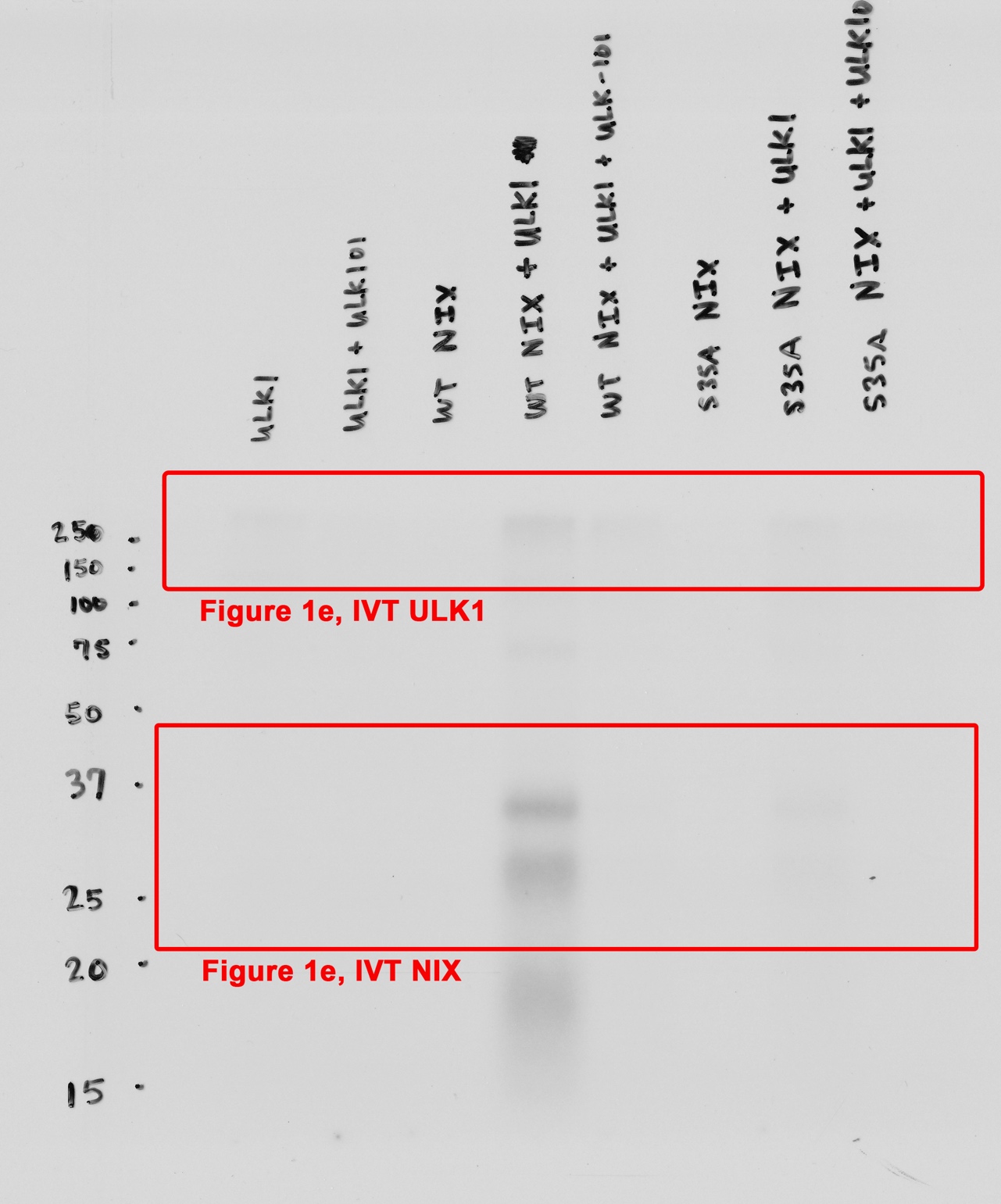


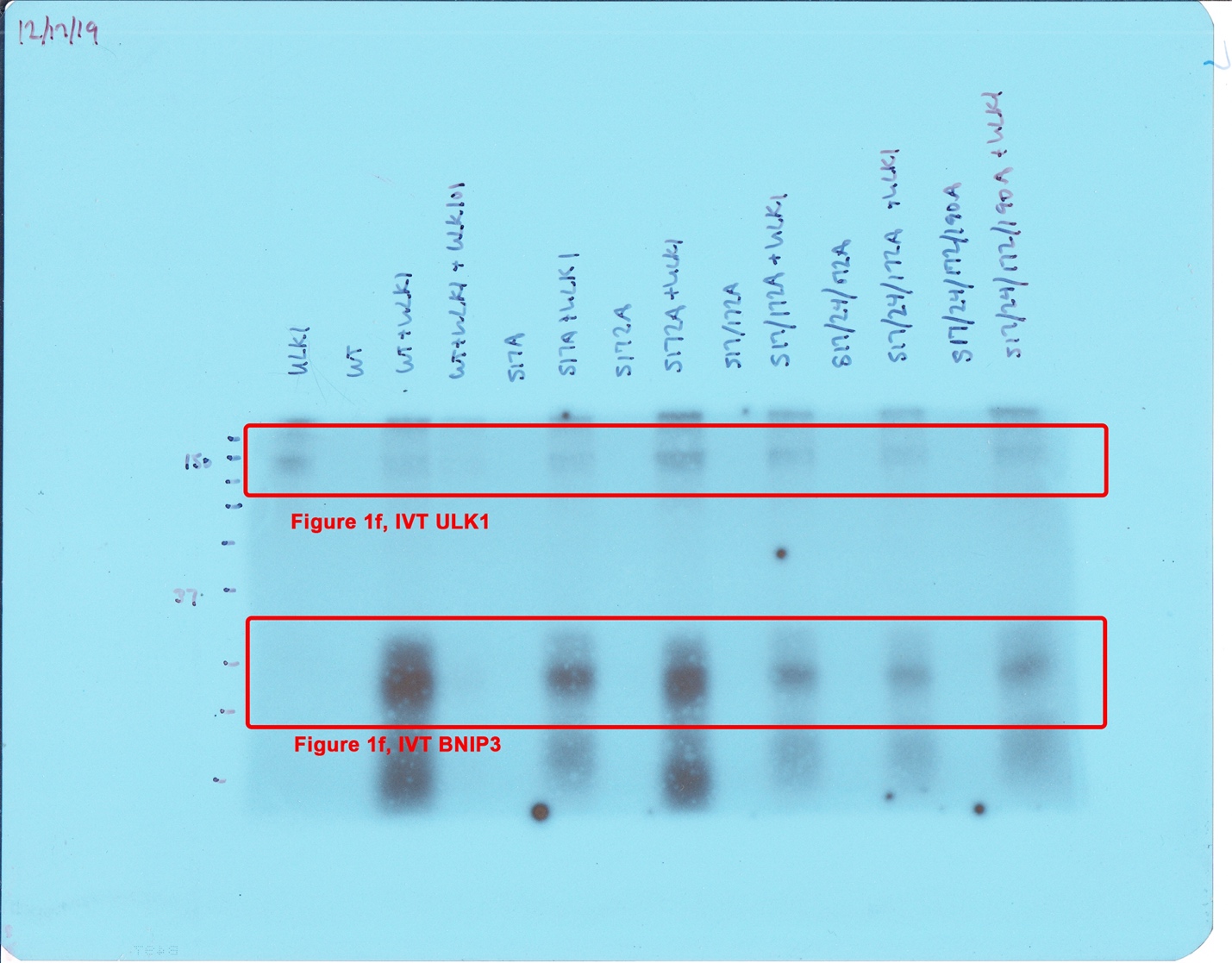


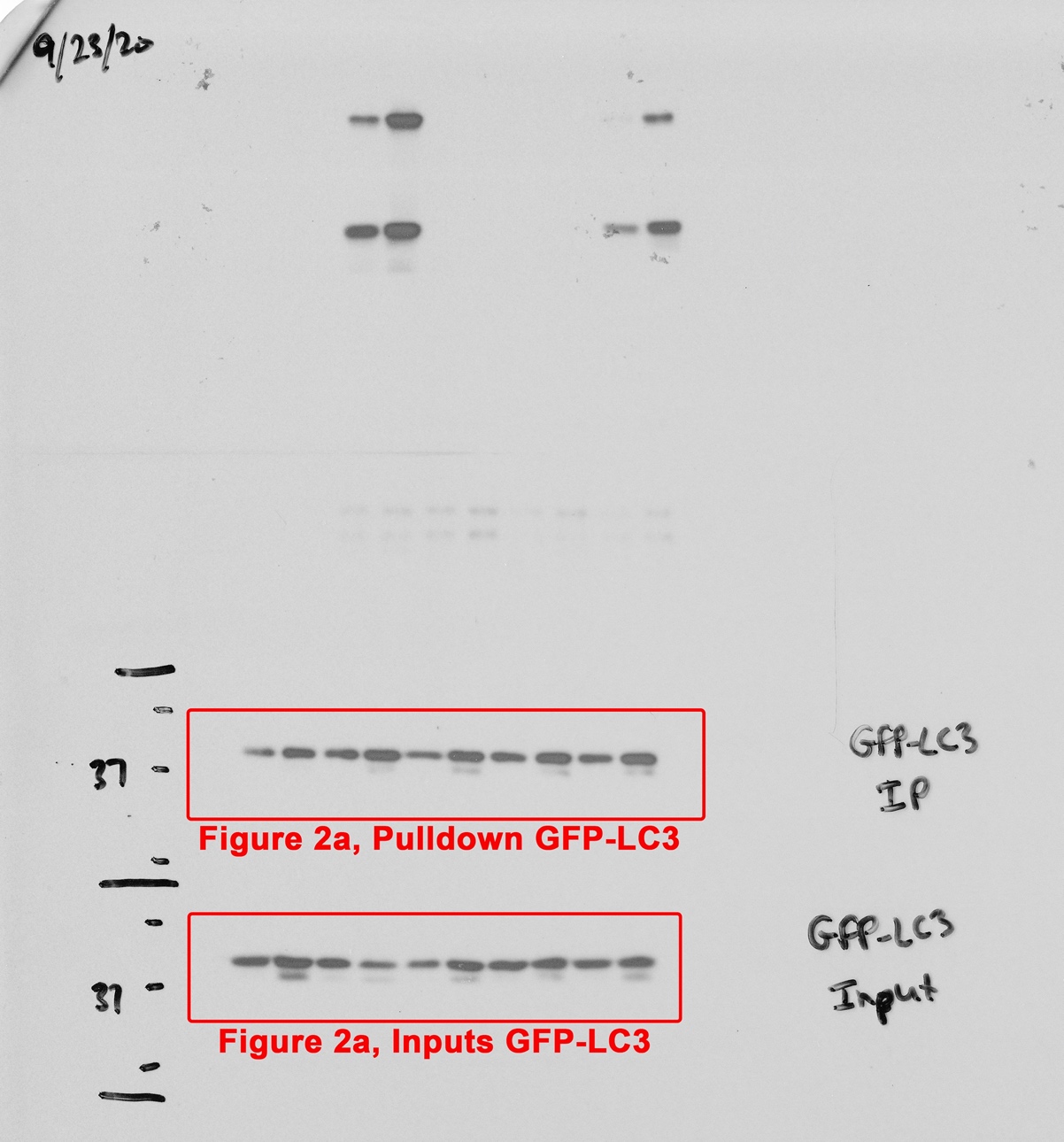


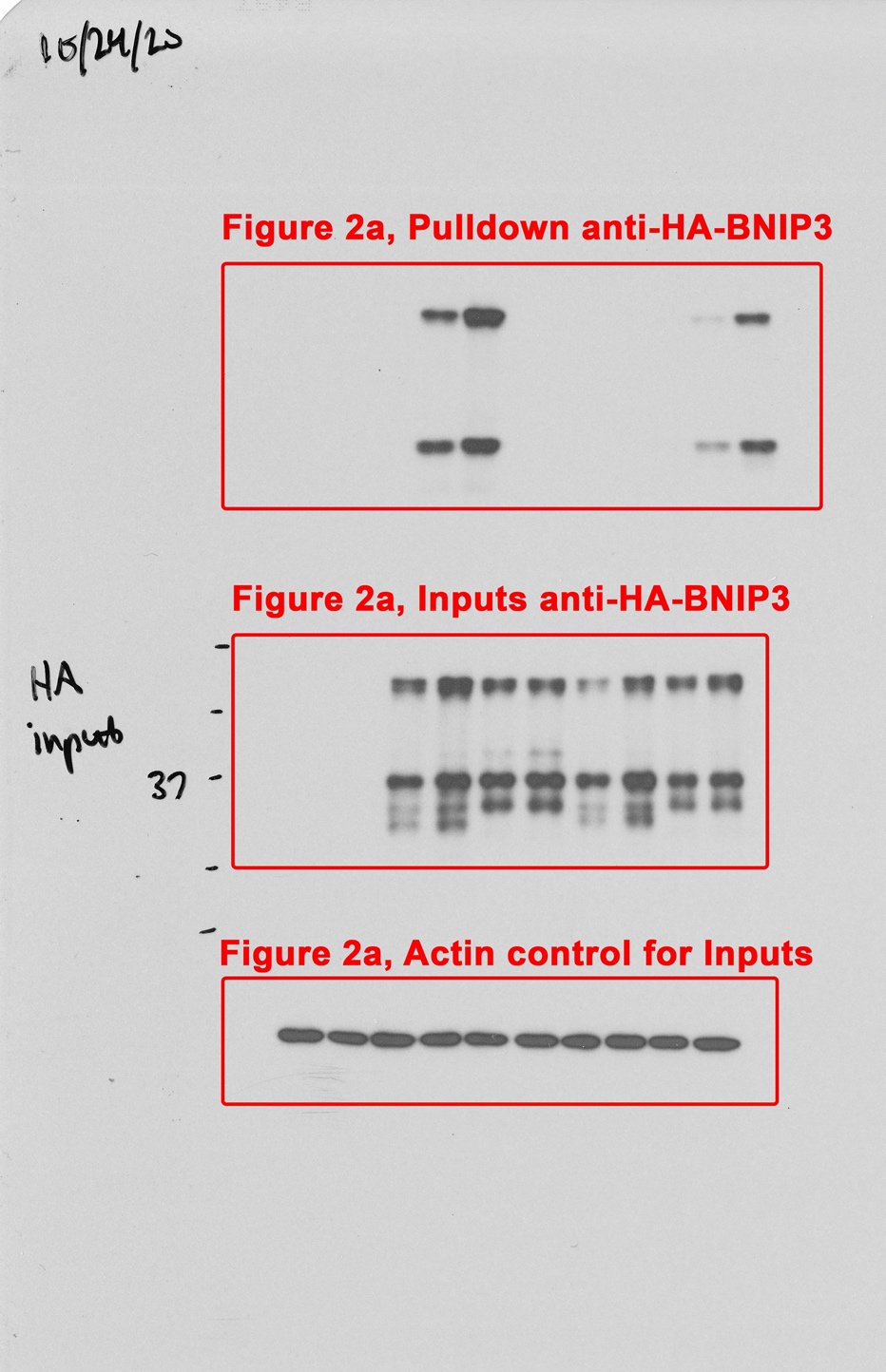


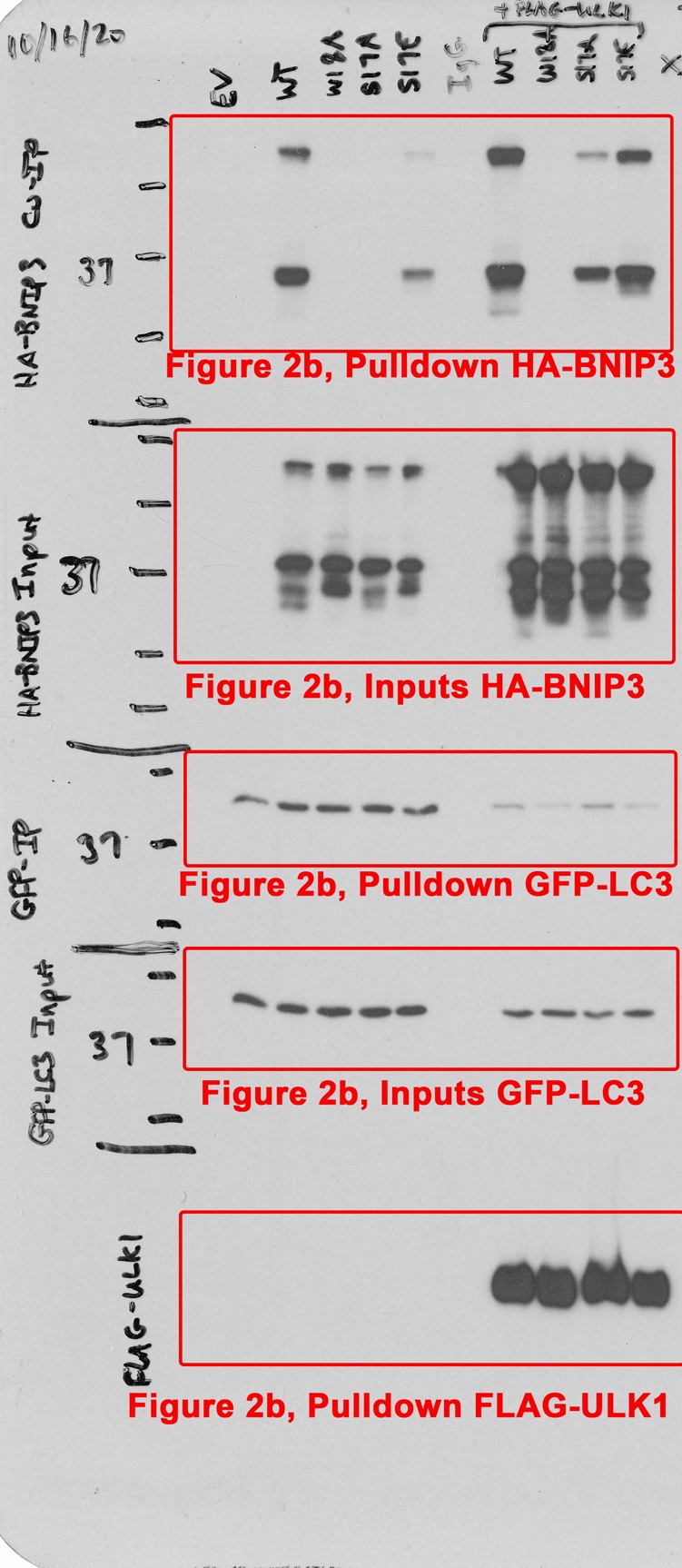


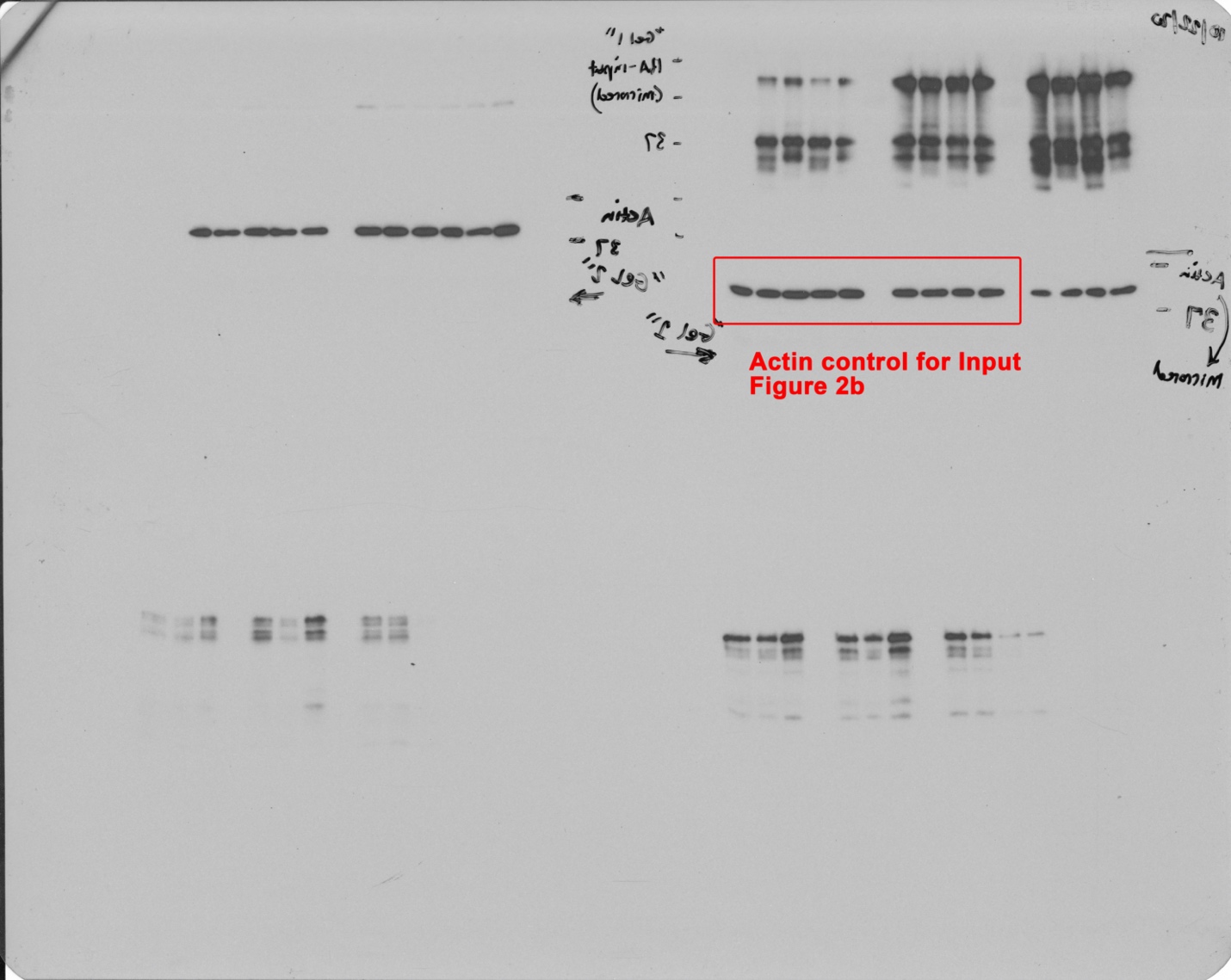


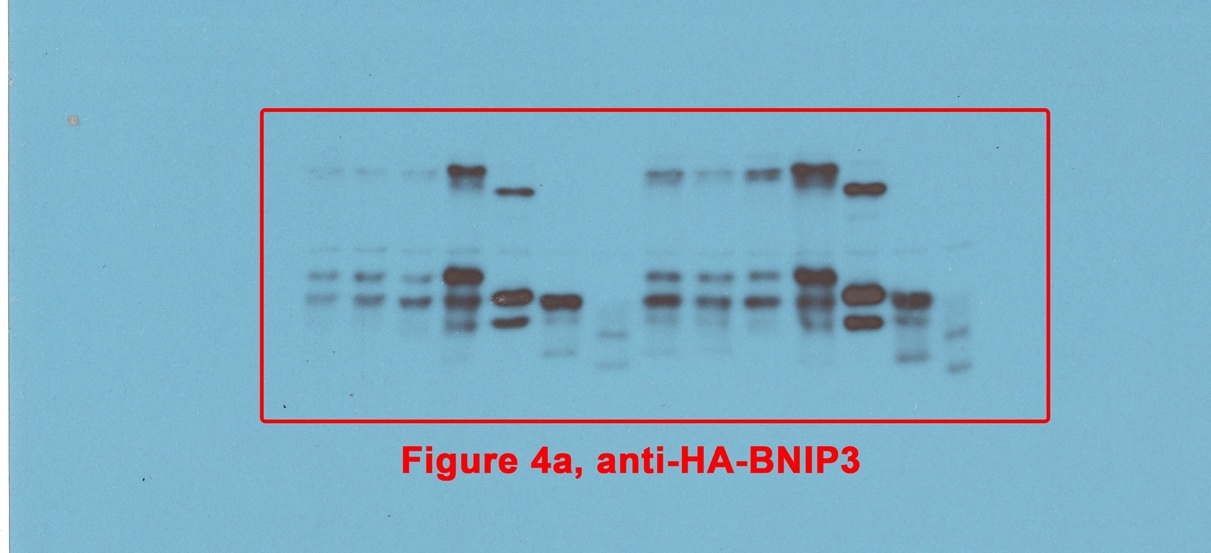


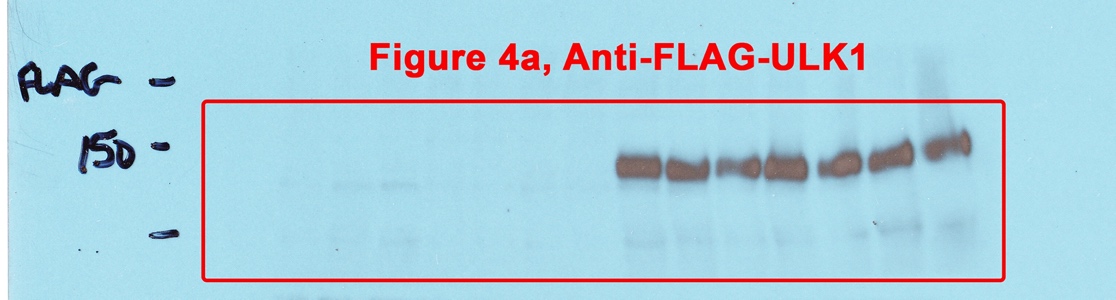


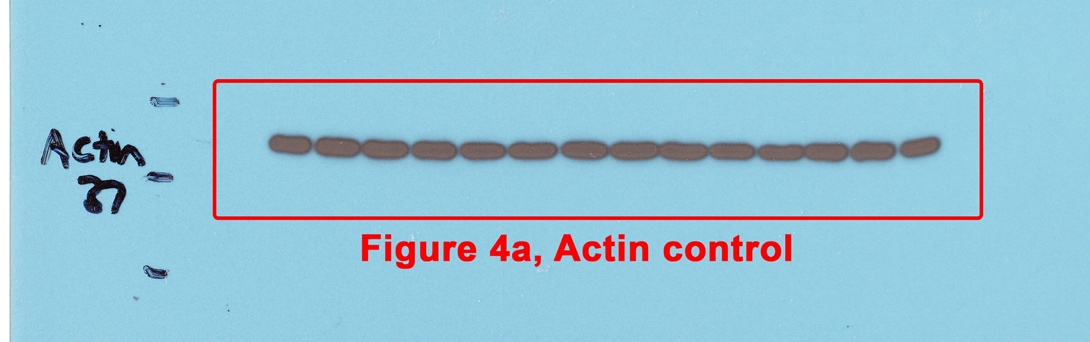


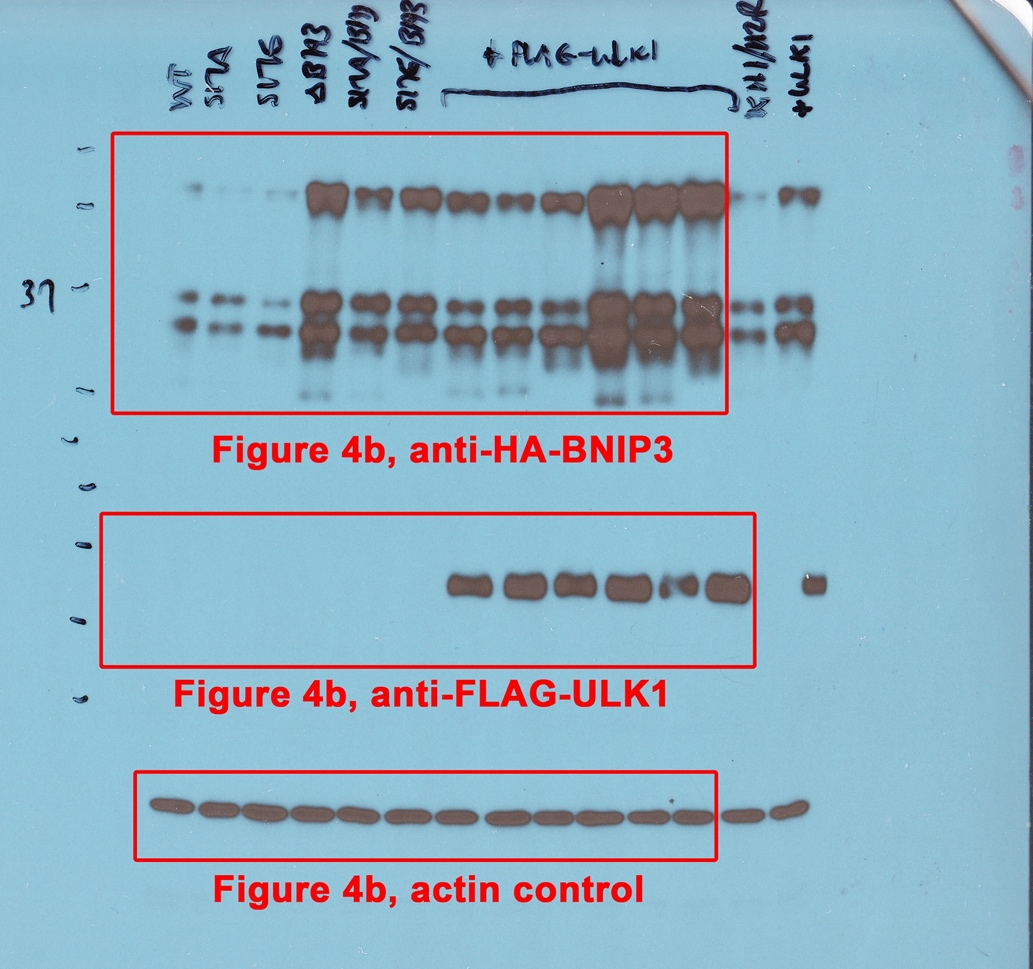


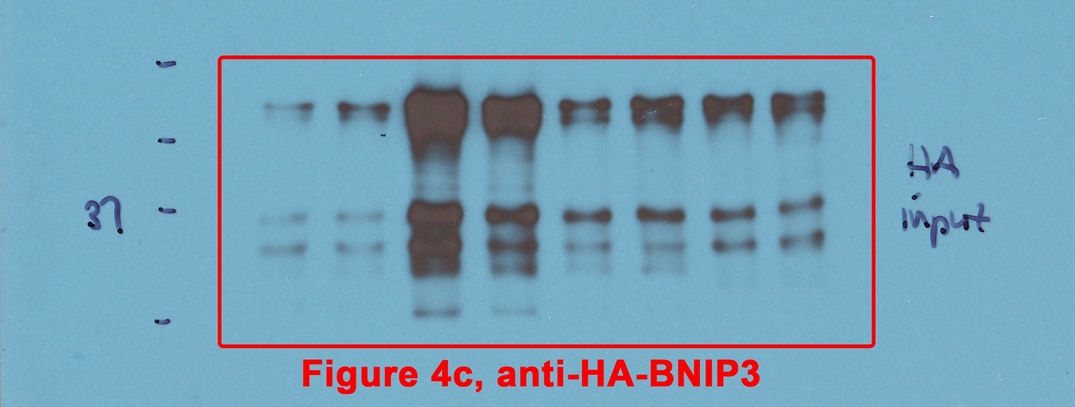


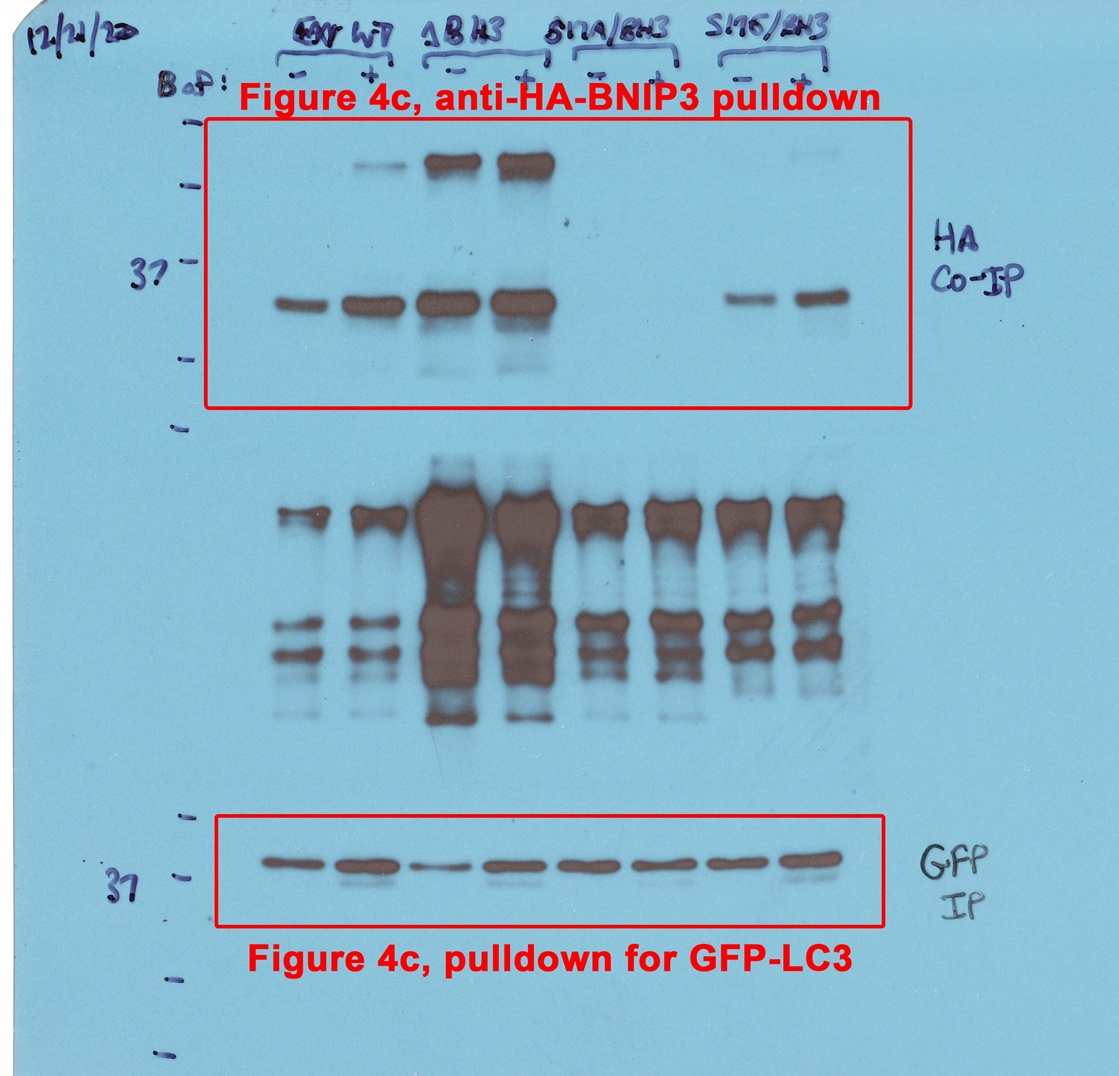

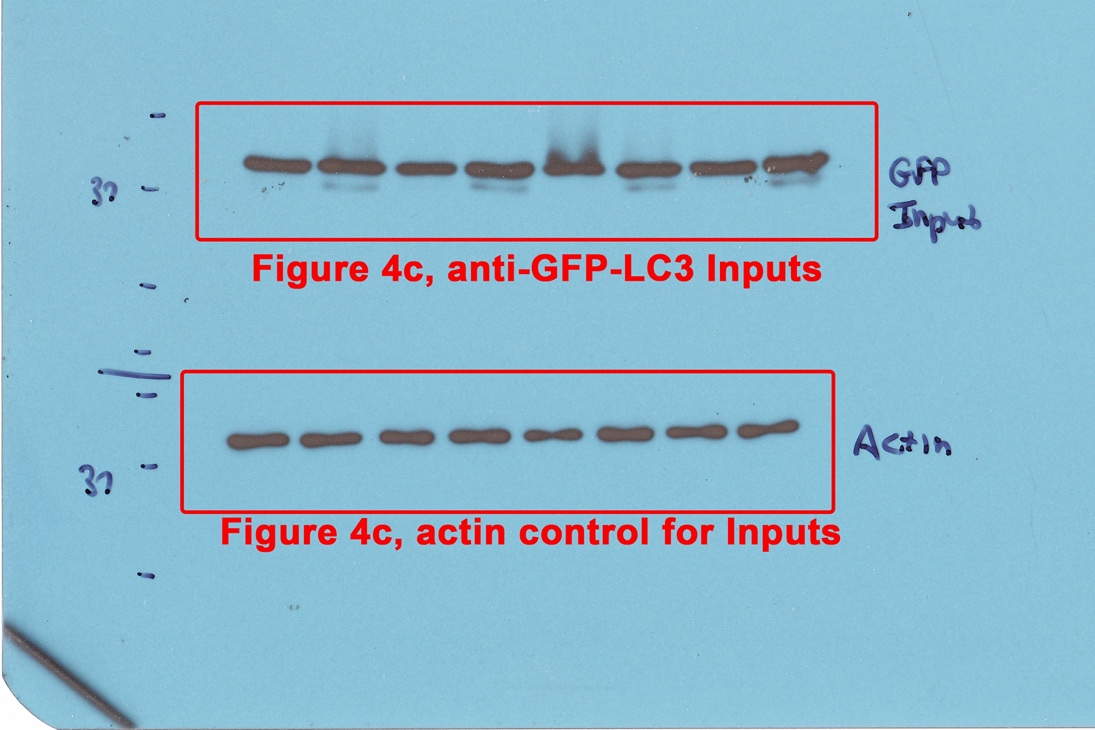


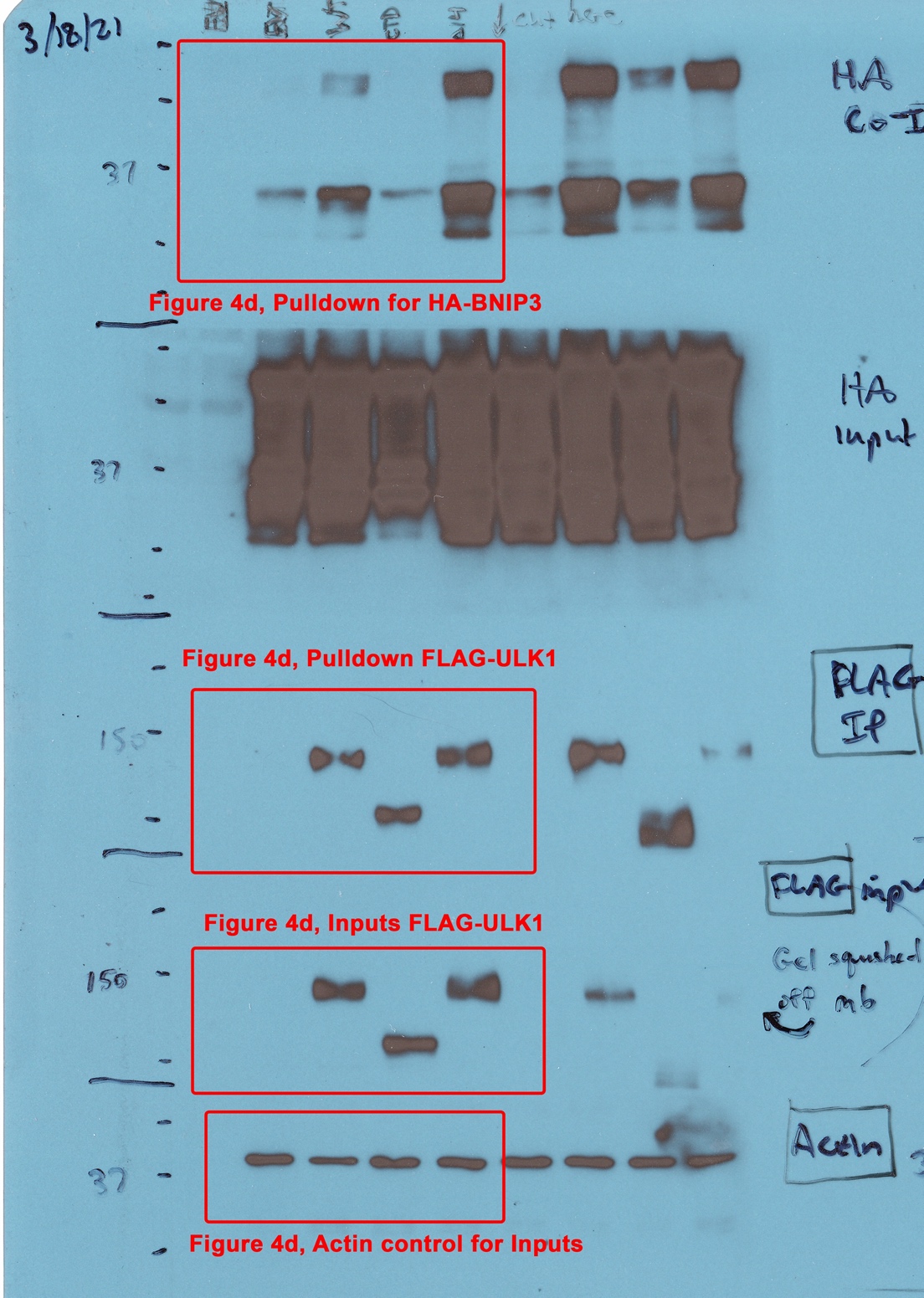


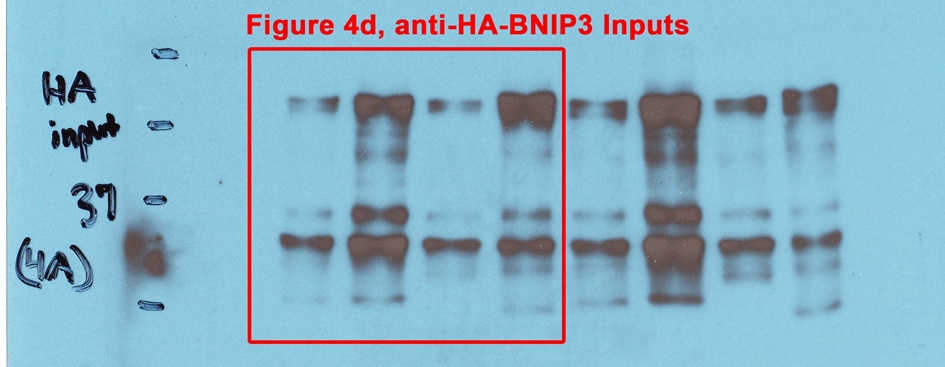


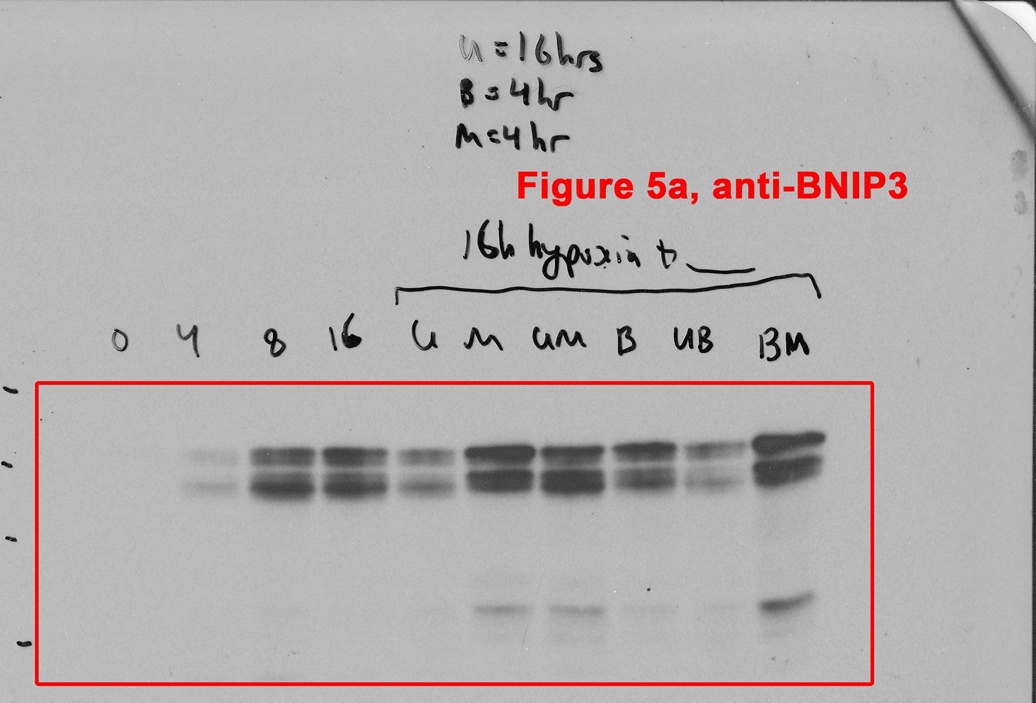


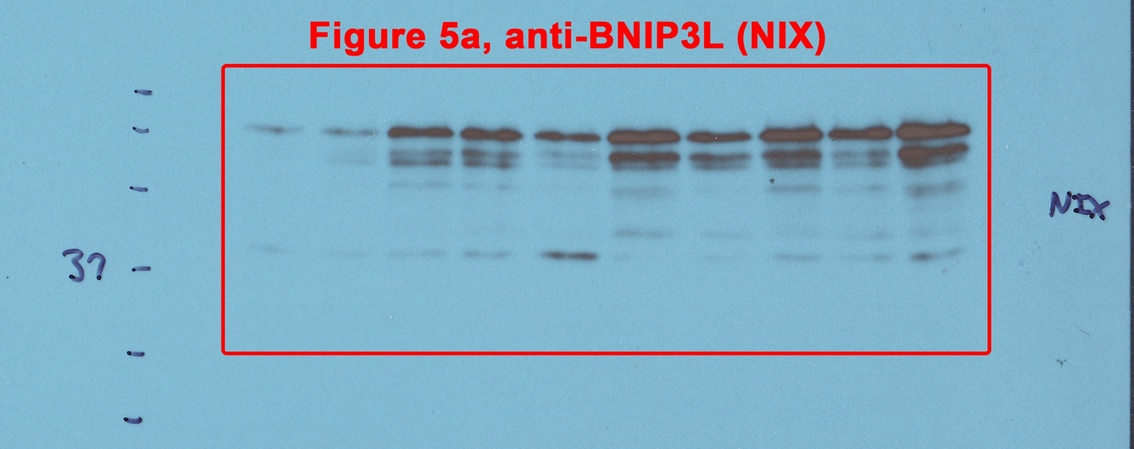


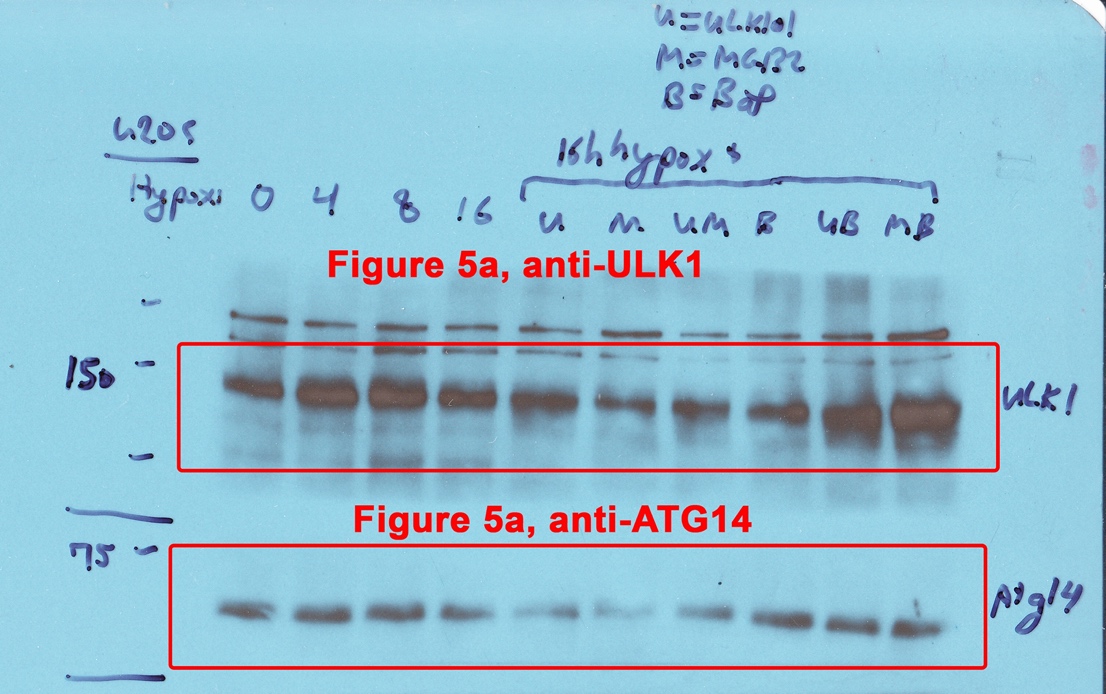


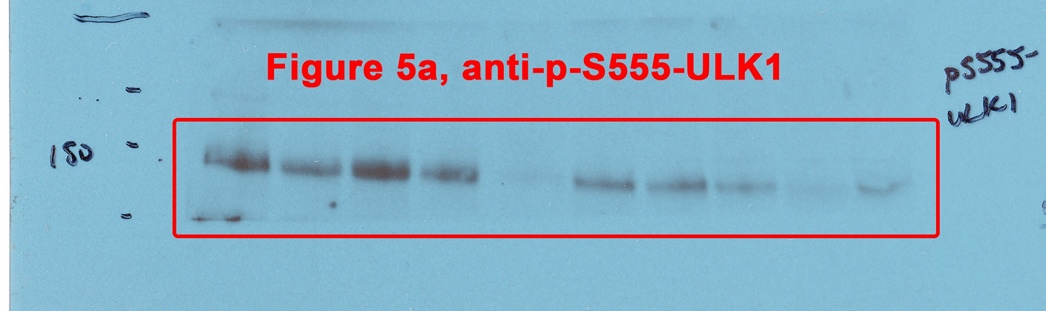


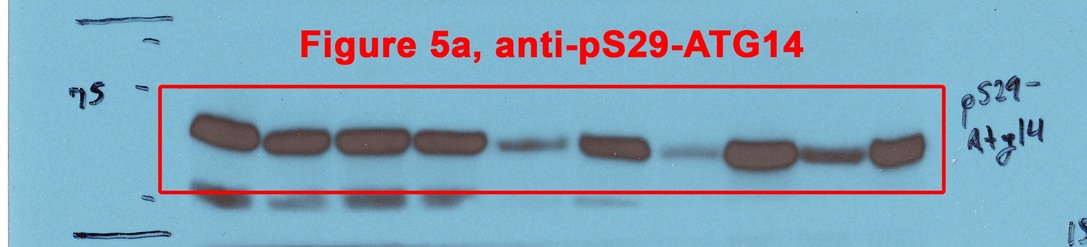


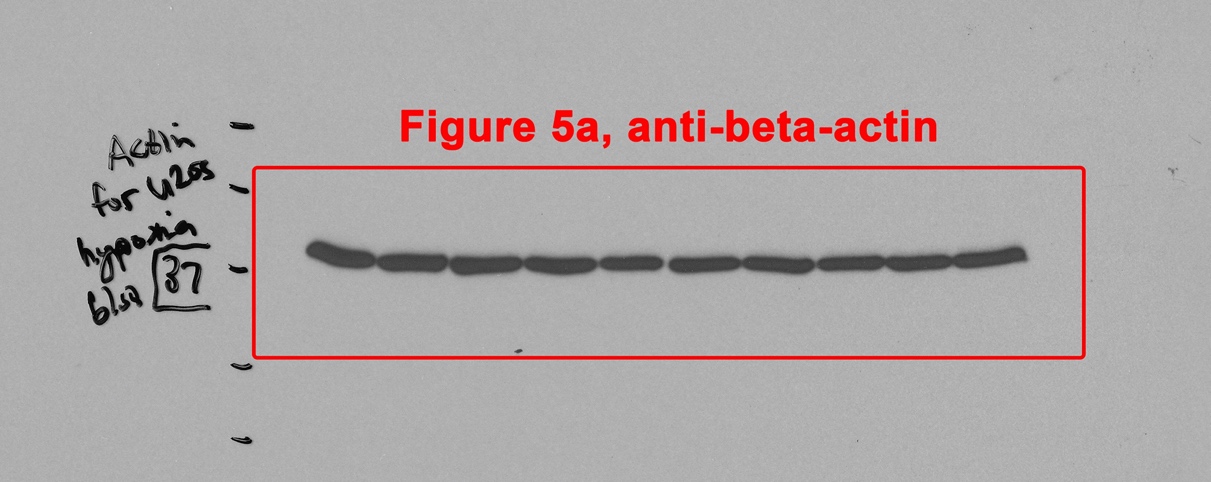


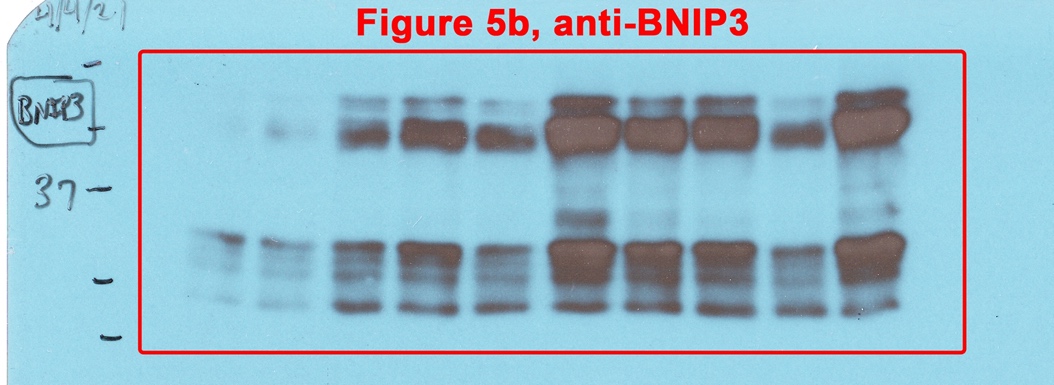


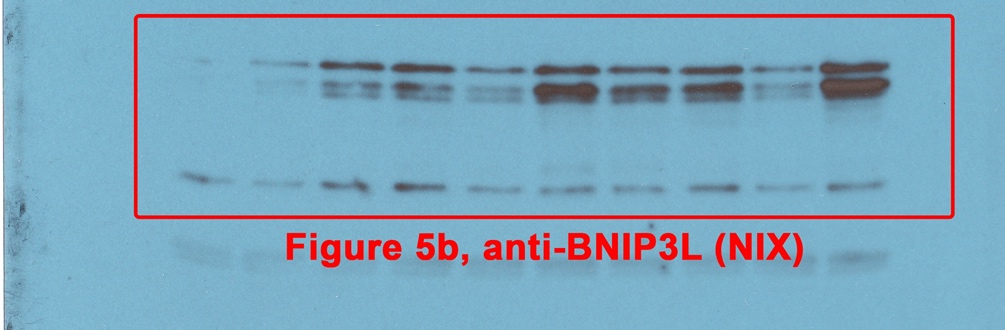


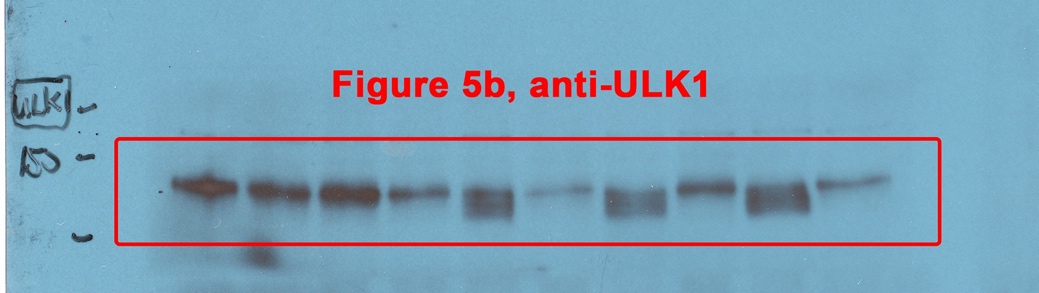


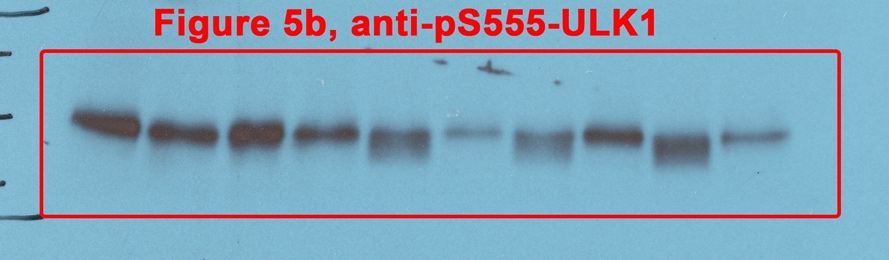


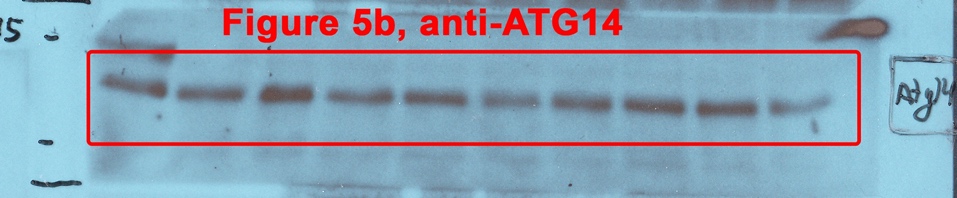


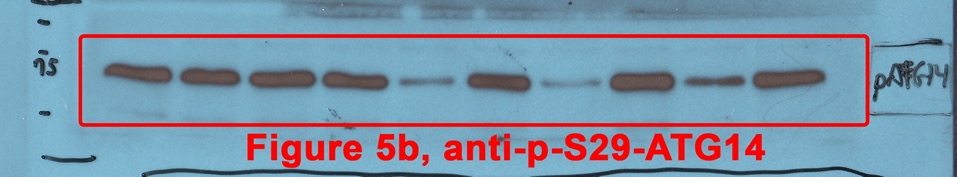


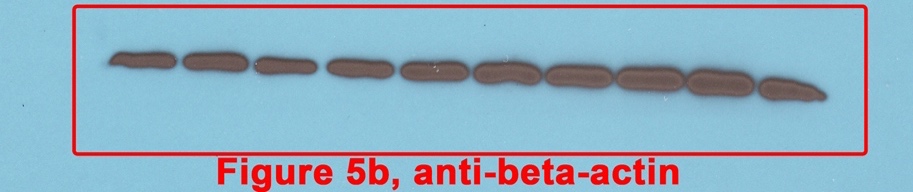


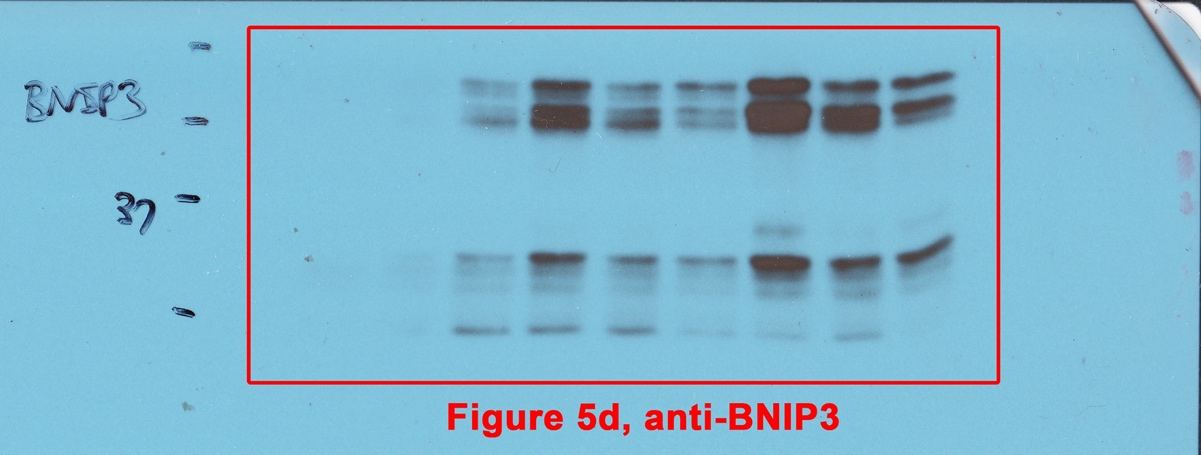


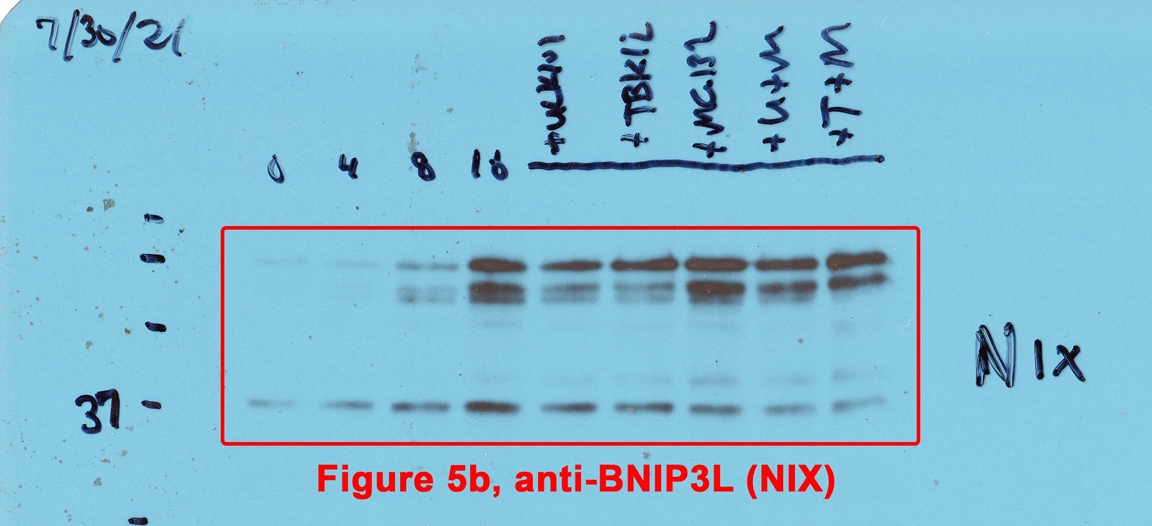


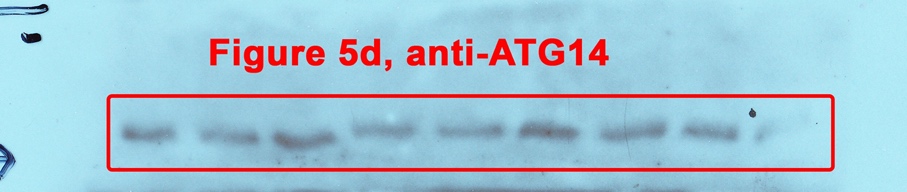


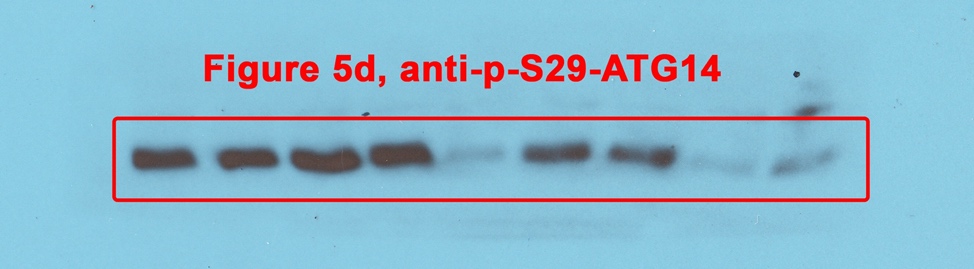


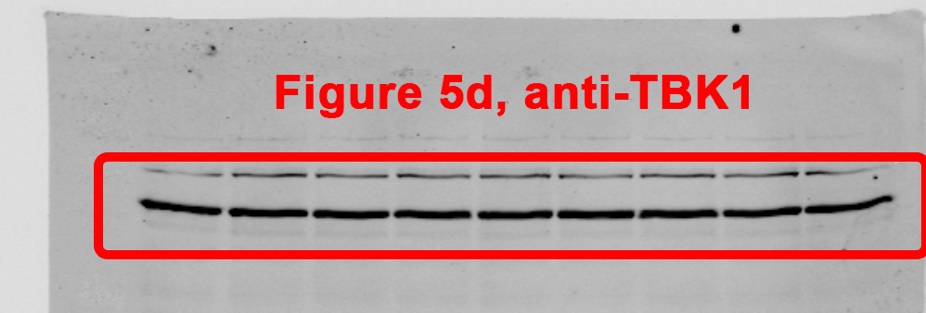


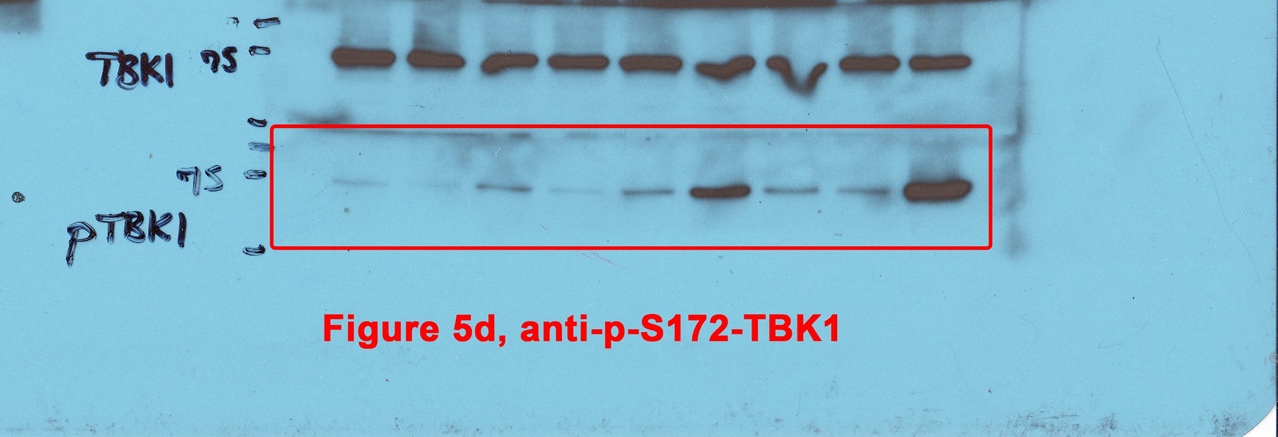


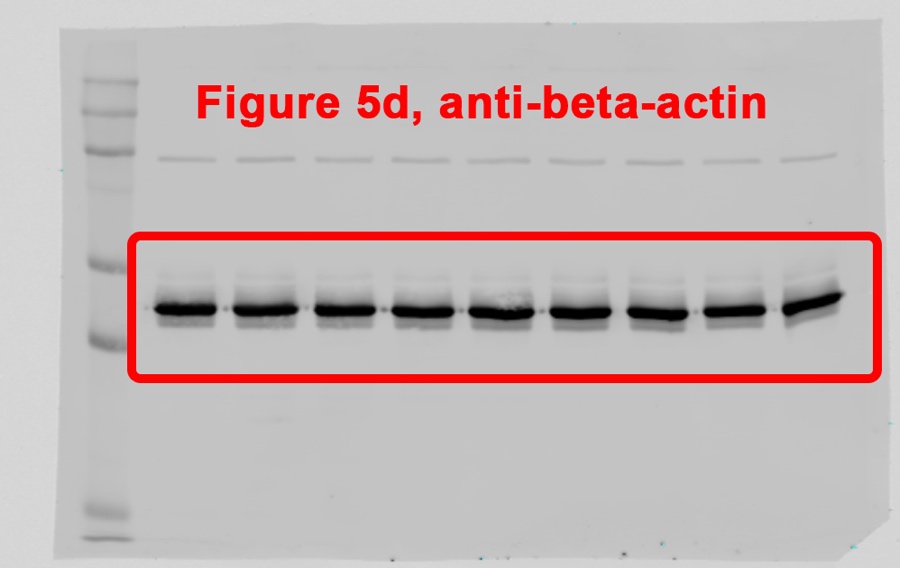


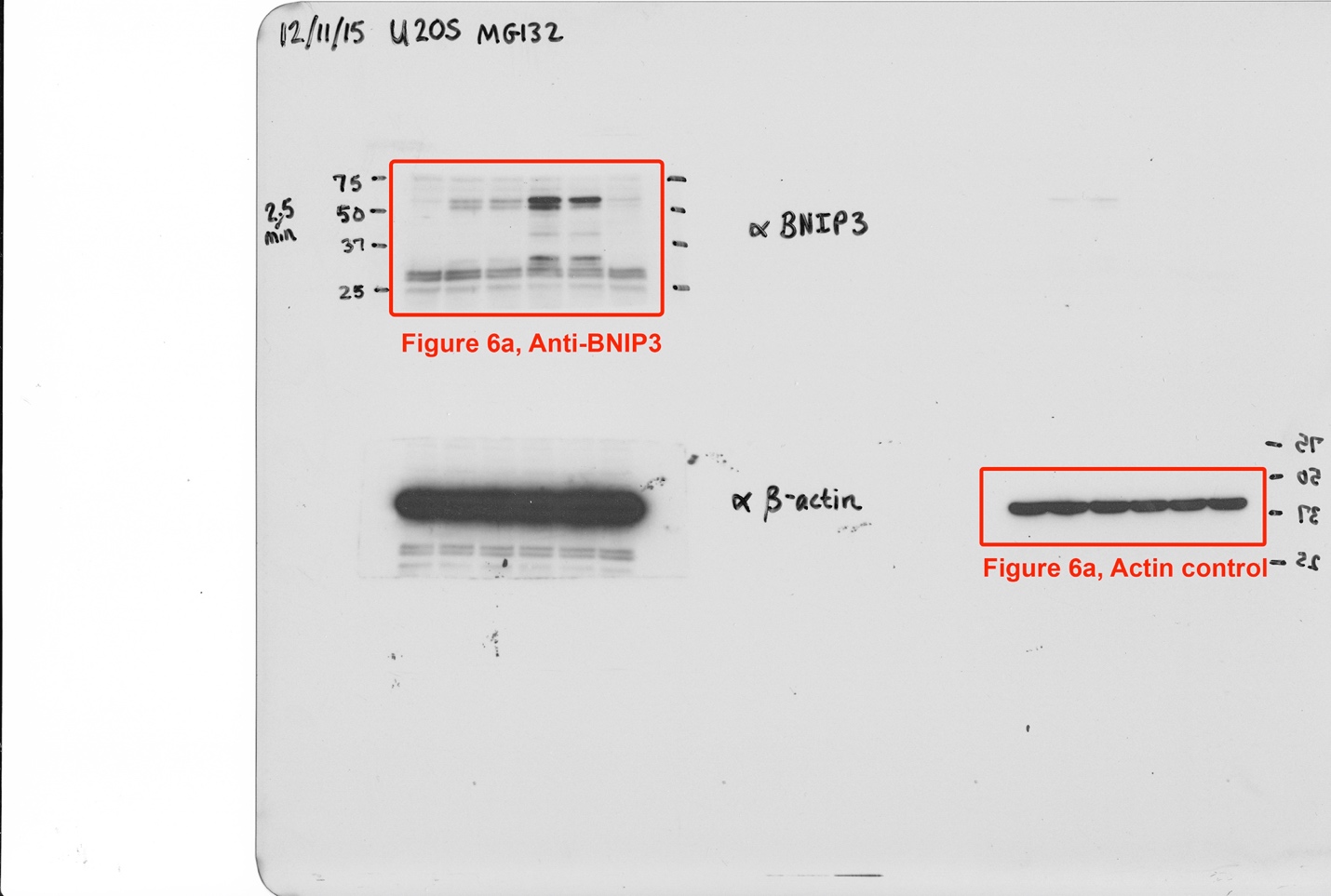


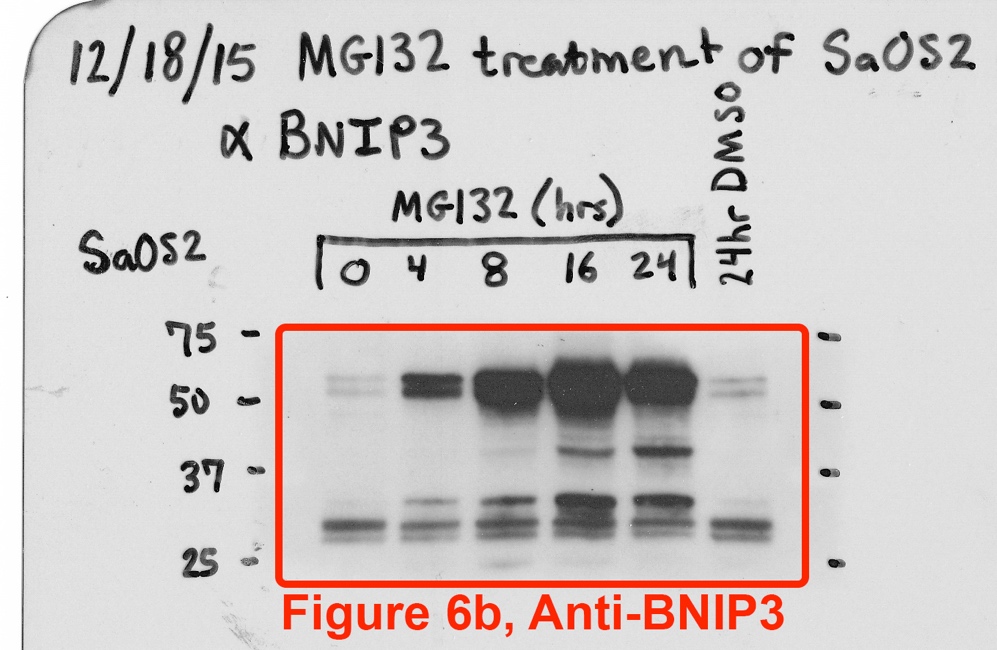


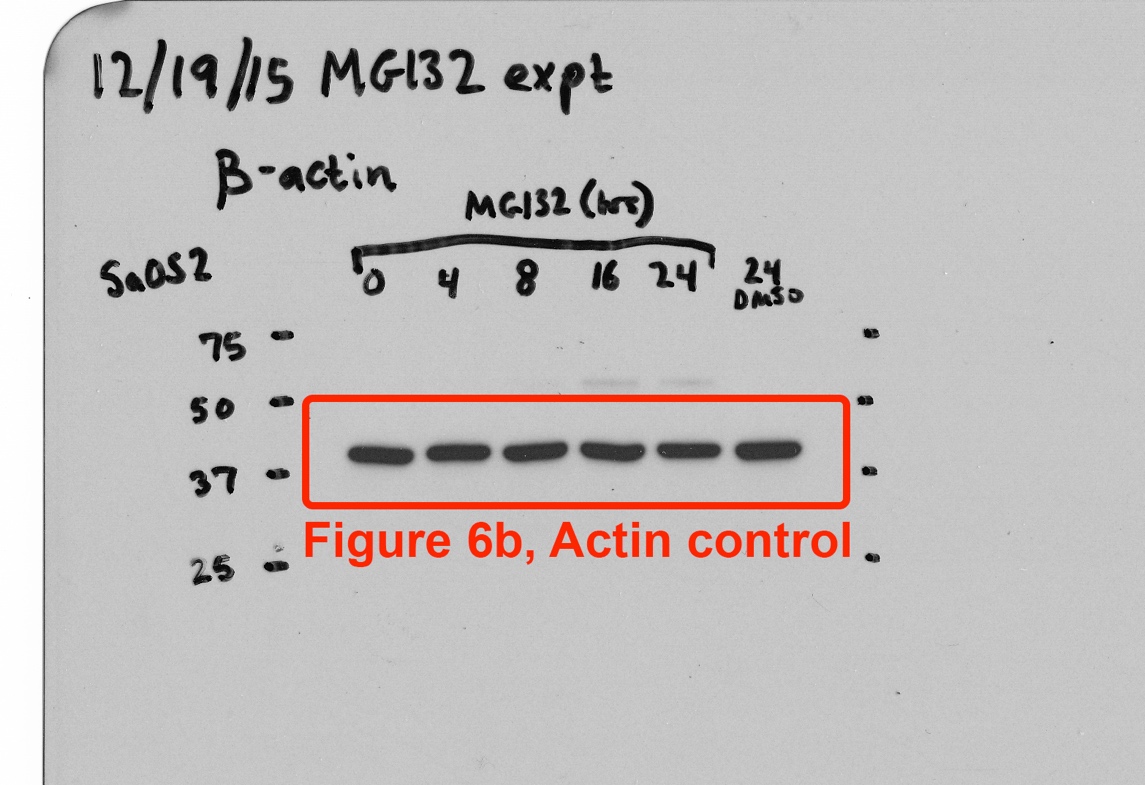


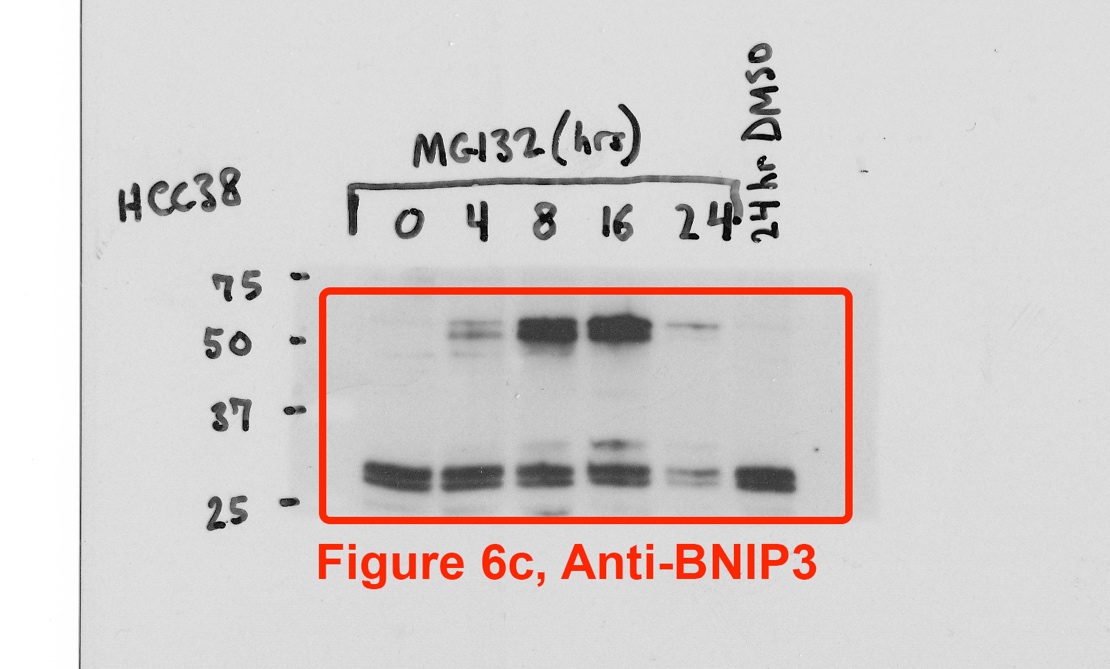


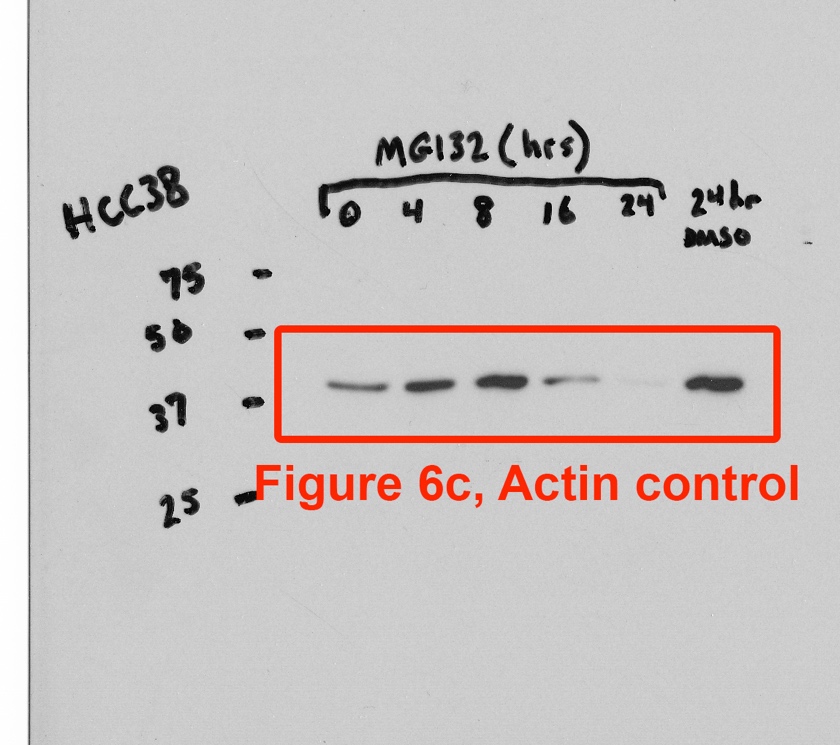


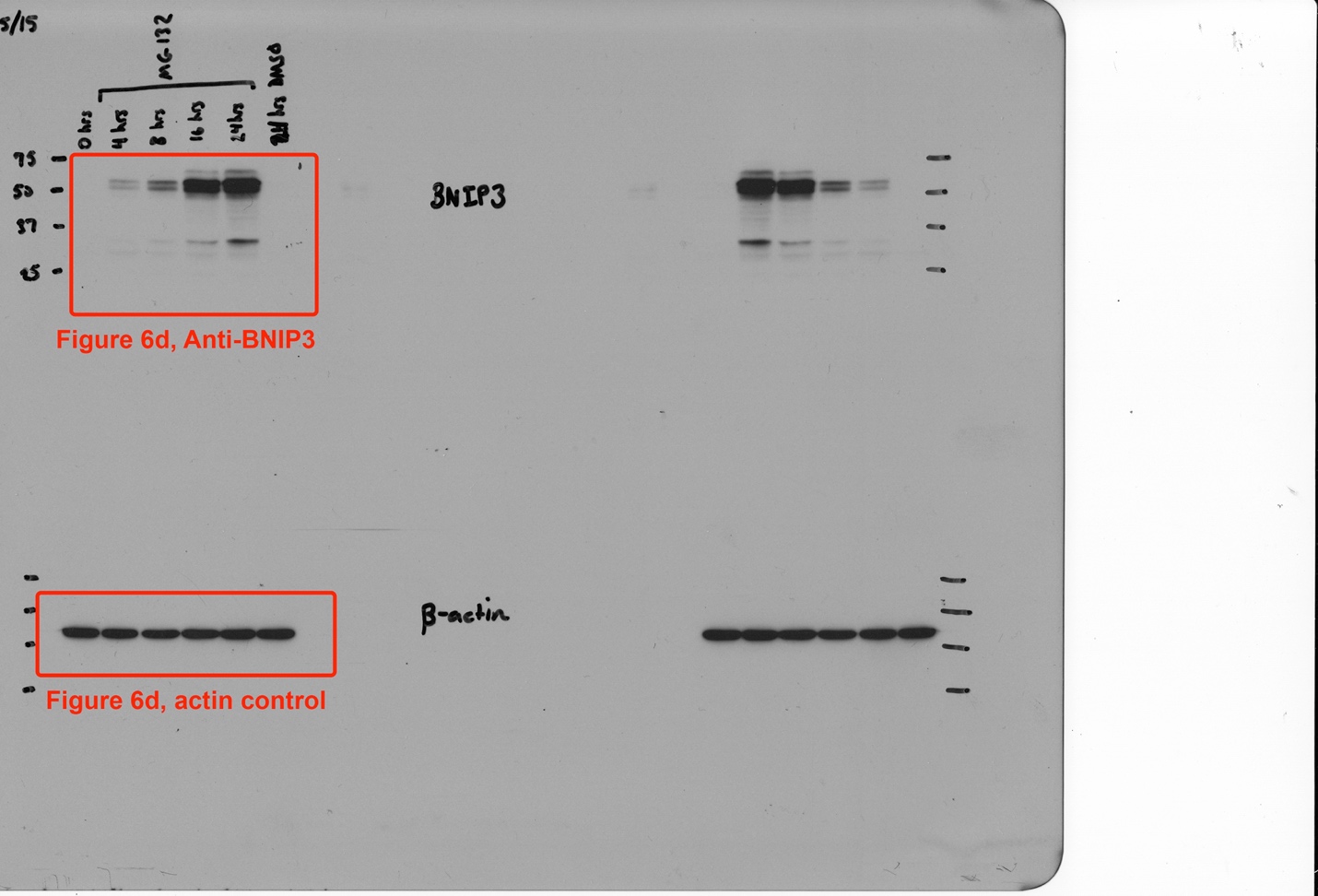


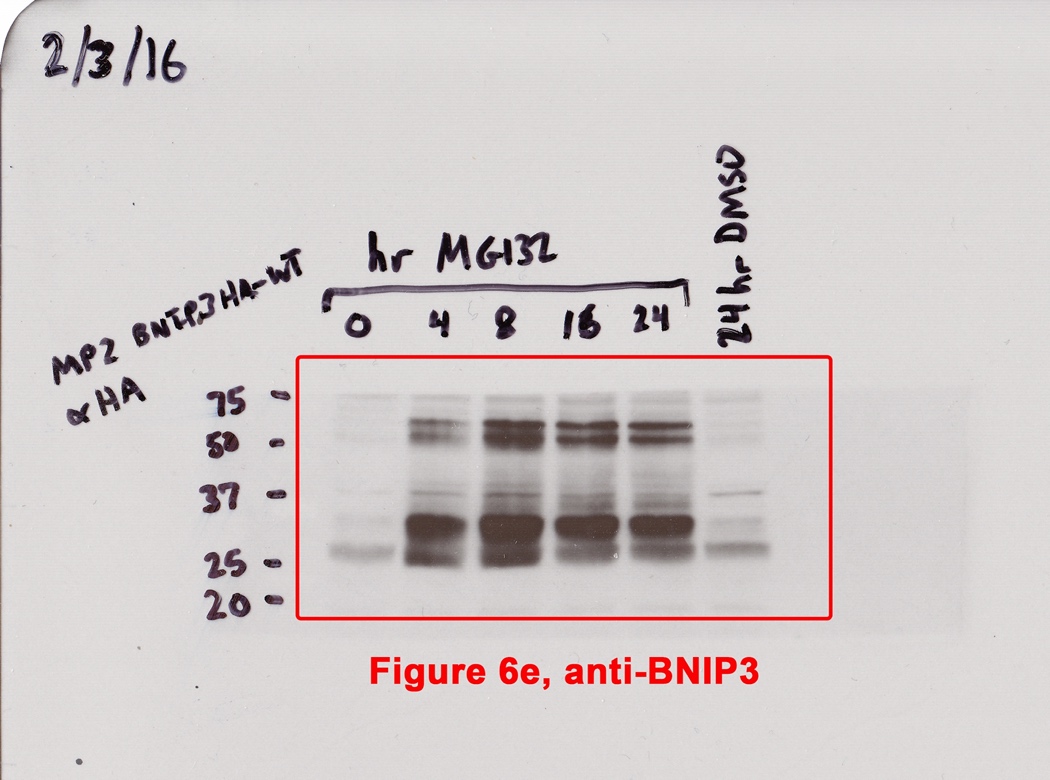


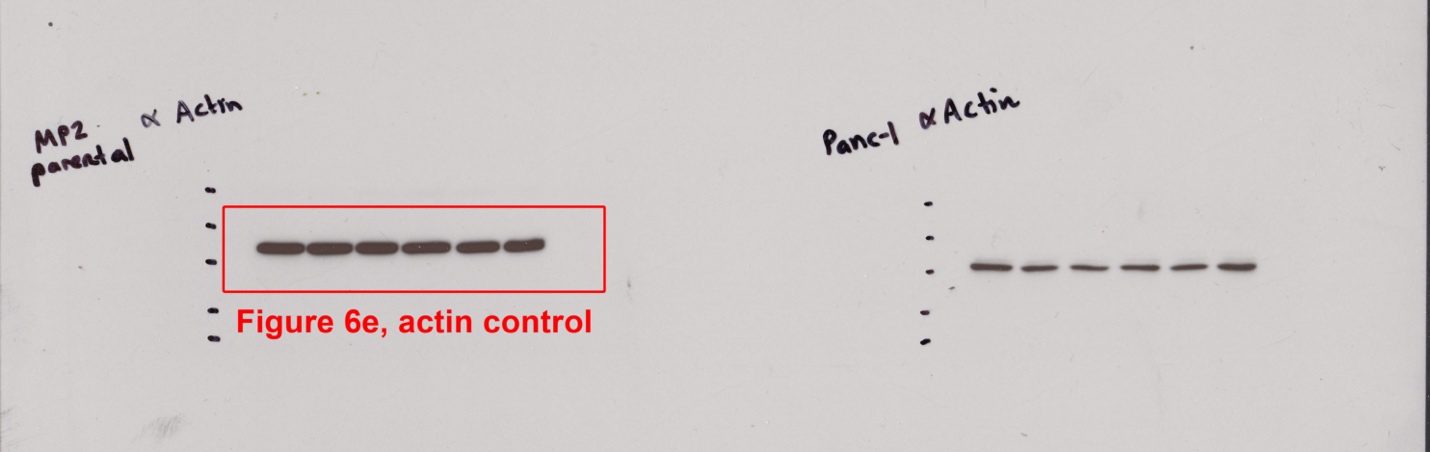


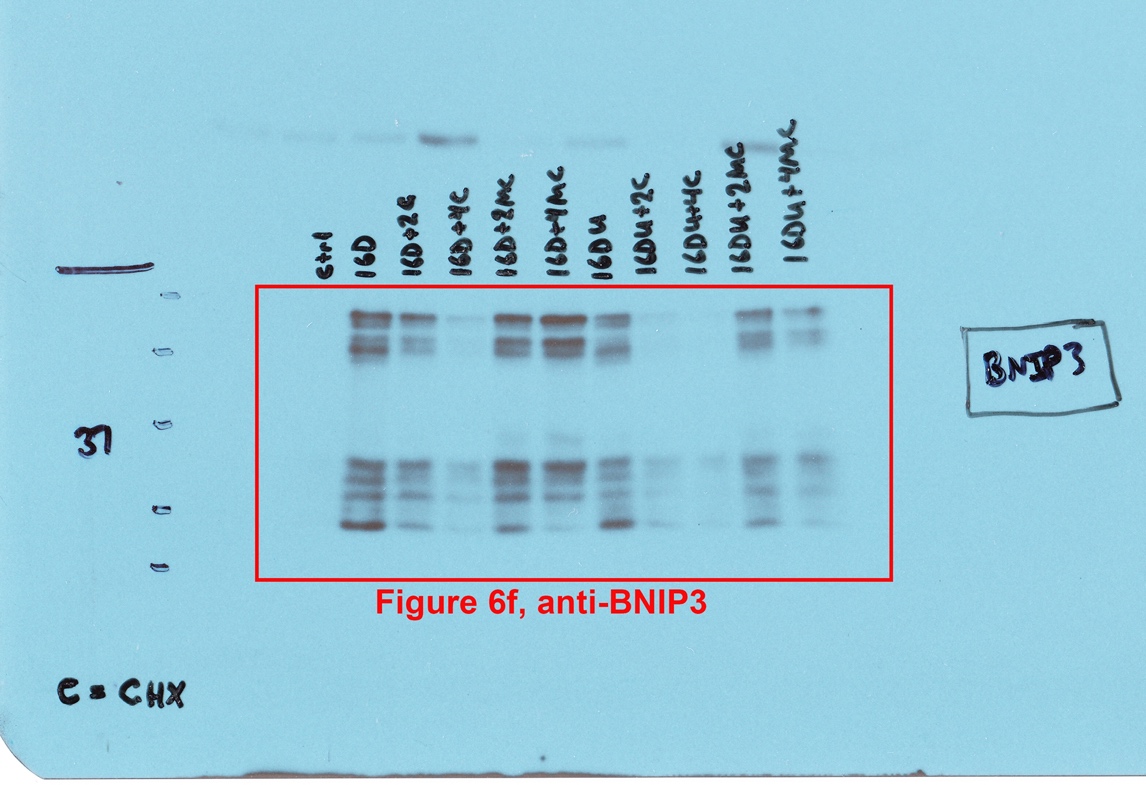


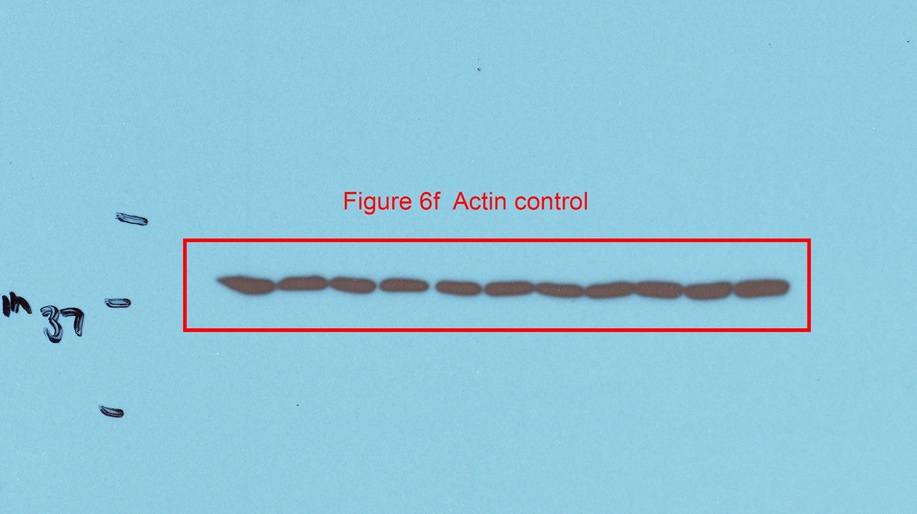

Supplement: Supplementary file 1 — Supplementary Information. [file 41598_2021_170_MOESM1_ESM.docx]
